# Comparative transcriptomic and proteomic kinetic analysis of adeno-associated virus production systems

**DOI:** 10.1007/s00253-024-13203-5

**Published:** 2024-06-19

**Authors:** Yu-Chieh Lin, Min Lu, Wen Cai, Wei-Shou Hu

**Affiliations:** Department of Chemical Engineering and Materials Science, University of Minnesota, 421 Washington Avenue S.E, Minneapolis, MN 55455-0132 USA

**Keywords:** rAAV, wtAAV, Transcriptomics, Proteomics, Manufacturing

## Abstract

**Abstract:**

Recombinant adeno-associated virus (rAAV) is a major gene delivery vehicle. We have constructed a stable rAAV producer cell line by integrating essential rAAV genome, viral and helper genes into the genome of HEK293 cell under the control of inducible promoters. Upon induction, the cell line produces transducing rAAV. To gain insight into enhancing rAAV productivity and vector quality, we performed a comparative transcriptomic and proteomic analysis of our synthetic cell line GX2 and two wild-type AAV (wtAAV) production systems, one by virus co-infection and the other by multi-plasmid transfection. The three systems had different kinetics in viral component synthesis but generated comparable copies of AAV genomes; however, the capsid titer of GX2 was an order of magnitude lower compared to those two wtAAV systems, indicating that its capsid production may be insufficient. The genome packaging efficiency was also lower in GX2 despite it produced higher levels of Rep52 proteins than either wtAAV systems, suggesting that Rep52 protein expression may not limit genome packaging. In the two wtAAV systems, VP were the most abundant AAV proteins and their levels continued to increase, while GX2 had high level of wasteful cargo gene expression. Furthermore, upregulated inflammation, innate immune responses, and MAPK signaling, as well as downregulated mitochondrial functions, were commonly observed in either rAAV or wtAAV systems. Overall, this comparative multi-omics study provided rich insights into host cell and viral factors that are potential targets for genetic and process intervention to enhance the productivity of synthetic rAAV producer cell lines.

**Key points:**

*• wtAAV infection was more efficient in producing full viral particles than the synthetic cell GX2.*

*• Capsid protein synthesis, genome replication, and packaging may limit rAAV production in GX2.*

*• wtAAV infection and rAAV production in GX2 elicited similar host cell responses.*

**Supplementary Information:**

The online version contains supplementary material available at 10.1007/s00253-024-13203-5.

## Introduction

Adeno-associated virus (AAV) derived-recombinant vectors are among the most commonly used vehicles for gene delivery in gene therapy. AAV is a small capsid virus with a 4.7 kb single-stranded DNA genome that contains *rep* and *cap* genes, which generate multiple transcripts and proteins through alternative splicing and the utilization of alternate start codons. The AAV gene encodes four nonstructural Rep proteins. The large Rep proteins Rep78/68 and the small Rep proteins Rep52/40 are indispensable for AAV genome replication and packaging, respectively (Snyder et al. 1993; King et al. 2001). VP1, VP2, and VP3 are encoded by the *cap* gene, which form the capsid that encapsidates the viral genome to form the virus particle (Maurer and Weitzman 2020; Muhuri et al. 2021). Assembly-activating protein (AAP) and membrane-associated accessory protein (MAAP), involved in AAV capsid assembly and secretion respectively, are also encoded by the *cap* gene (Sonntag et al. 2011; Elmore et al. 2021; Galibert et al. 2021).

In gene therapy applications, the *rep* and *cap* genes flanked between two inverted terminal repeats (ITRs) are replaced with the gene of interest (GOI) to become a recombinant AAV (rAAV) vector. Thus, the rAAV viral particle consists of a capsid that encases a genome containing only the therapeutic GOI. Upon AAV entry into a target cell, the capsid dissociates in the cytoplasm and the viral genome enters the nucleus where second-strand DNA synthesis occurs, allowing transcription to proceed and gene expression to commence (Wang et al. 2019). AAV does not normally integrate into the host cell genome, but the small single-stranded ( +) and (-) genomes can form concatemer to become circular which may persist in nucleus episomally for a long period of time and continue to express GOI (Wang et al. 2019).

rAAV DNA integrates into the genome of the target cell at a very low frequency, is replication incompetent, and is generally regarded as safe for in vivo use (Naso et al. 2017). This is in contrast to the genome-integrating vectors such as lenti and retro viral vectors, which have been reported to give rise to oncogenic mutations, are primarily used for ex vivo gene delivery (Bulcha et al. 2021).

AAV infection of a cell results in latent infection unless it is co-infected with a helper virus, such as adenovirus (Ad). Adenoviral E1A, E1B, E2A, E4orf6, and VA RNA have been identified as helpers that are essential for or facilitate AAV replication. E1A and E2A, a DNA binding protein (DBP), activate the AAV p5 and p19 promoters to control the expression of AAV Rep proteins; both E1B and E4orf6 promote AAV second-strand synthesis; VA RNA prevents the E4orf6/E1B complex from degrading AAV capsids and Rep52 (Meier et al. 2020). A classical method for the production of AAV is the co-infection of cells with Ad and AAV (Zeltner et al. 2010). The method requires live preparations of both AAV and Ad. To circumvent the need for two viruses, a commonly used alternative is to use two plasmid vectors, one containing the AAV genome and the other encoding essential Ad helper genes. Transient transfection of these two plasmids results in the production of AAV while no Ad virus is produced. Similarly, transfection of plasmids containing the rAAV genome, Ad helper genes, and *rep* and *cap* genes in trans has become a classical method for rAAV production (Clement and Grieger 2016). The produced rAAV vector is replication-incompetent and is free of other viruses, as is required for gene therapy vectors. This method is widely used to produce rAAVs for research and clinical trials.

However, multi-plasmid transient transfection-based rAAV production faces challenges. The production requires the generation of large quantities of multiple plasmids that may subject to lot-to-lot variability (Srivastava et al. 2021). Furthermore, in transfection, different cells would receive varying copy numbers of each plasmid, resulting in potentially vastly different levels of viral components and heterogeneity of viral particles. It has been reported that vectors produced by plasmid transfection have a high proportion of empty particles (Clement and Grieger 2016). The heterogeneity of plasmid distribution in cells complicates attempts at quality control by turning plasmid composition.

We have developed a platform of a stable rAAV producer cell line for robust manufacturing. Integrated in the genome of HEK293-based producer cell line were a Genome Module encoding a green fluorescent protein (GFP) reporter gene flanked by AAV2 ITRs, a Replication Module consisting of Rep68 coding sequence (CDS) and adenoviral helper DBP and E4orf6 CDS, and a Packaging Module containing a AAV2 intron-less *cap* gene and the Rep52 CDS (Supplemental Fig. S1a) (Lu et al. 2024a). In the Genome module, *GFP* was driven by an inducible LacSwitch to suppress its expression in the producer cells (Lu et al. 2024a). The Rep68, DBP, and E4orf6 CDS were driven by the inducible TetOn promoter, while AAV2 intron-less *cap* and the Rep52 CDS were driven by the inducible CumateSwitch promoter. Through a series of design-build-test cycle and employing transcriptomic and quantitative proteomic analysis, the rAAV-producing GX cell lines were established and shown to have comparable encapsidated rAAV productivity to that of commonly used triple plasmid transient transfection (Lu et al. 2024a). By manipulating the induction profile, the kinetics of genome replication and capsid formation could be tuned to adjust the full particle content (Lu et al. 2024a).

rAAV is generally administered at a very high dose. For efficient manufacturing, further improvements in the productivity of the synthetic cell line are needed. To this end, we sought to compare the performance of a synthetic cell line with a benchmark AAV production system. We postulate that a system producing wild-type AAV (wtAAV) would be a good benchmark. wtAAV is thought to have evolved an optimal strategy for capsid assembly, as well as replication and packaging of viral genomes (Zeltner et al. 2010) and thus provide a biological bound for production of rAAV. We chose two wtAAV production systems for comparison, one with co-infection of adenovirus type 5 (Ad5) and wtAAV and a second one with transfection of two plasmids carrying wtAAV genome and the essential adenoviral helper genes (Supplemental Fig. S1b).

We characterized the transcriptome, performed absolute quantification (AQUA) of viral proteins by targeted quantitative proteomics, and conducted tandem mass tag (TMT)-based proteomics on the three rAAV and wtAAV production systems. In this report, we highlighted key findings on viral transcript and protein dynamics, and globally compared cellular responses to viral replication, assembly, and packaging.

## Materials and methods

### Cells and cell culture

HEK293 cells obtained from Cell Biolabs Inc. (San Diego, CA, USA) were maintained in Dulbecco's Modified Eagle Medium (DMEM containing 4.5 g/L glucose; Gibco, ThermoFisher, Waltham, MA, USA) supplemented with 10% fetal bovine serum (FBS; Gibco, ThermoFisher, Waltham, MA, USA) and antibiotic/antimycotic (Gibco, ThermoFisher, Waltham, MA, USA) in T-flasks at 37 °C in a 95% humidity and 5% CO_2_ air atmosphere. The rAAV production cell GX2 was constructed as described before (Lu et al. 2024a) and briefly described in Supplemental Fig. S1a. GX2 cells were maintained in our laboratory.

### AAV production

Case A: For rAAV production using GX2, cells were seeded at 3.75 × 10^5^ cells per well in a 6-well plate (7.8 × 10^4^ cells/cm^2^). After 16 h, the culture medium was replaced with induction medium containing 10 μg/mL doxycycline and 90 μg/mL cumate (Sigma-Aldrich, St. Louis, MO, USA). Case B: HEK293 cells were seeded in 7.5 × 10^5^ cells per well in a 6-well plate (1.56 × 10^5^ cells/cm^2^) and grown for 16 h before co-infection of wtAAV2 (#VR-680, ATCC, Manassas, VA, USA) at multiplicity of infection (MOI) 100 (based on vector genome (VG)/mL) and Ad5 (#VR-5, ATCC, Manassas, VA, USA) at MOI 10 (based on TCID_50_ (50% tissue culture infectious dose)). Case C: HEK293 cells were cultured under the same conditions as in Case B for 16 h, then were transfected with wtAAV2 genome-carried plasmid pAV1 (#37,215, ATCC, Manassas, VA, USA) and adenoviral helper gene-carried pHelper (Cell Biolabs, San Diego, CA, USA) using Transporter 5 transfection reagent (Polysciences, Warrington, PA, USA). For all three cases, cells were harvested at various time points for cell lysis for isolation of rAAV/wtAAV, intracellular DNA, RNA, and proteins.

### AAV preparation and quantification

The AAV sample preparation for assay of encapsidated VG by quantitative polymerase chain reaction (qPCR) and capsids by enzyme-linked immunosorbent assay (ELISA) were as described previously (Lee et al. 2022). For AAV VG titer determination using qPCR, the primer pairs targeting AAV2 ITRs (Aurnhammer et al. 2012) and cargo gene *GFP* (Lee et al. 2022) were used. The AAV2 reference materials (RS-AAV2-FL, Charles River Laboratories, Hollister, CA, USA) were included as a positive control and used as a reference standard to determine the VG titer. The capsid titer was measured using AAV2 Titration ELISA kit (PRATV, PROGEN, Wayne, PA, USA). The full particle content was determined by dividing the VG by the capsid titer (Grimm et al. 1999). Intracellular DNA was extracted using Quick-DNA/RNA kits (Zymo Research, Orange, CA, USA) and subsequently used for titration of intracellular total AAV genome (TG) copies, including free and encapsidated viral genomes, via qPCR. The copies of TG were calculated in reference to the copy number of *GPR15* in the genome of HEK293 cell. All the primers used for qPCR analysis were synthesized by Integrated DNA Technologies Corp. (Coralville, IA, USA) and their sequence were listed in Supplemental Table S1.

### RNA sequencing and data analysis

RNA was extracted from the harvested cells using the RNeasy Mini kit (QIAGEN, Germantown. MD, USA). The preparation of the library, the read length and depth, the sequencing platform, the read mapping and counting, and the transcripts per million (TPM) calculation have been described previously (Lee et al. 2022). In detail, the reads were mapped to the human genome (GRCh38) with the addition of (i) Ad5 *E1A*, *E1B* and three integrated transposon vector sequences for Case A, (ii) AAV2 genome (NC_001401.2), Ad5 genome (AC_000008.1) and pHelper sequence for Case B and C. Transcript expression was assessed using Cufflinks and reported as TPM. Differential expression analysis between different sample groups was performed using the EdgeR package in R (Robinson et al. 2010). Transcripts with a value of false discovery rate (FDR) *p* < 0.05 and a log_2_ fold change (FC) > 1 or < -1 were considered significantly differentially expressed. Functional enrichment analysis of the differentially expressed genes (DEGs) and functional annotation clustering were performed using the web-based tool DAVID (Dennis et al. 2003). Functional class data of Gene Ontology (GO) and Kyoto Encyclopedia of Genes and Genomes (KEGG) databases were used (Ashburner et al. 2000; Kanehisa and Goto 2000; Huang et al. 2009). Functional classes with a value of unadjusted *p* < 0.05 were considered significantly enriched in DAVID. Pre-ranked gene set enrichment analysis (GSEA) using all expression data as input was performed using the biological process of GO terms (GOBP) database (Subramanian et al. 2005). Normalized enrichment score (NES) > 1 or < -1, normalized *p*-value < 0.05, and *q*-value < 0.25 were used as criteria to identify significantly enriched functional classes in GESA. All raw RNA-seq data were deposited in the NCBI database (BioProject: PRJNA1076915).

### Quantitative proteomics using tandem mass tags and data analysis

The cell pellet was washed twice with ice-cold phosphate-buffered saline (PBS) and reconstituted in lysis buffer (7.0 M urea, 2.0 M thiourea, 0.4 M Tris, 40 mM chloroacetamide, 10 mM Tris (2-carboxyethyl) phosphine (TCEP), 20% acetonitrile). Protein samples were quantified using Bradford method and then digested into peptides with trypsin. The quantitative proteomics using 16-plex TMTs was performed by University of Minnesota Center for Metabolomics and Proteomics. The details of the procedure of TMT labeling, data acquisition using Orbitrap Fusion (Thermo Fisher Scientific, Waltham, MA, USA), database searching for protein detection, and relative protein quantification using Proteome Discoverer™ Software (Thermo Fisher Scientific, Waltham, MA, USA) were described previously (Yang et al. 2022). Proteins with a value of unadjusted *p* < 0.05 were considered differentially expressed for all comparisons in this study. Functional enrichment assays and functional annotation clustering with differentially expressed proteins in each comparison were conduct using DAVID with GO and KEGG databases (Ashburner et al. 2000; Kanehisa and Goto 2000; Huang et al. 2009). Pre-ranked GSEA was also performed with expression data, relative abundance ratio, of all the detected proteins using the GOBP database (Subramanian et al. 2005). The criteria for determining significantly enriched functional classes in DAVID and GSEA were the same as those used for RNA-seq. The mass spectrometry proteomics data are available via ProteomeXchange with identifier PXD050484.

### Targeted quantitative proteomics analysis

Protein sample preparation, parameters used for acquiring parallel reaction monitoring (PRM)-based targeted mass spectrometry data, and raw data processing on Skyline were described in detail previously (MacLean et al. 2010; Lee et al. 2022; Lu et al. 2024a). The ratio of light to heavy peptides was calculated from the peak areas, and the ratios were then used for the determination of endogenous protein concentrations. Human beta-actin proteins encoded by *ACTB* (UniProt accession: P60709) were also quantified and used as an internal control. The sequence of the heavy isotope-labeled peptides used in this study were listed in Supplementary Table S2. Viral protein copies per cell were calculated based on the assumption that the total protein of a HEK293 cell is 360 pg. The mass spectrometry proteomics data are available via ProteomeXchange with identifier PXD050484.

## Results

### Different viral component kinetics in three cases

**Fig. 1:**
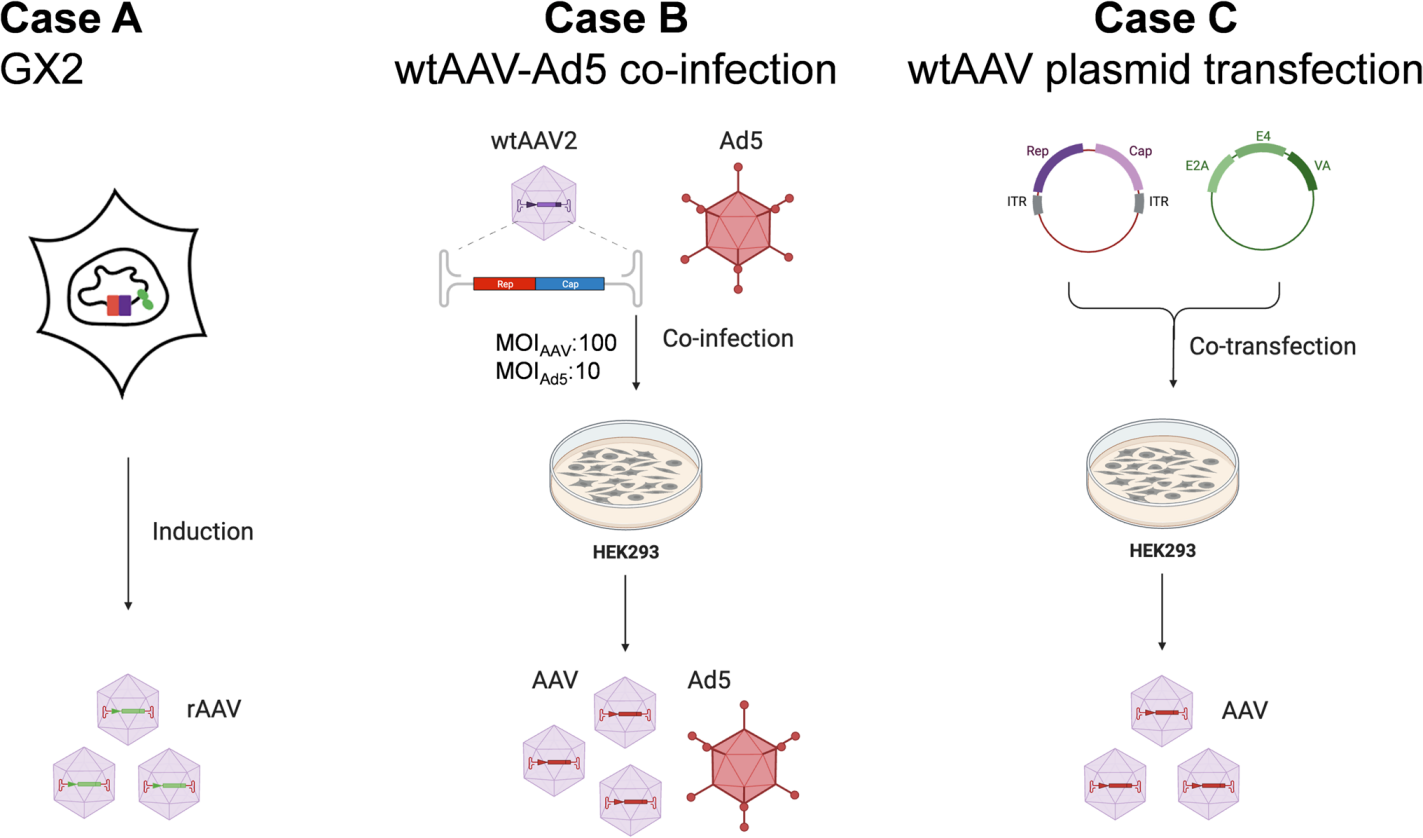
Schematic diagram of three AAV production systems. Case A: rAAV2 production by synthetic producer cell line GX2 upon induction with doxycycline and cumate. Case B: wtAAV2 production by HEK293 cells co-infected with wtAAV2 and Ad5. Case C: wtAAV2 production by HEK293 cells co-transfected with wtAAV2 genome-carried plasmid pAV1 and adenovirus helper gene-carried plasmid pHelper. This figure was created with BioRender.com

We compared rAAV production in a synthetic HEK293-derived cell line, GX2, which could produce rAAV2 upon induction **(**Case A**)** (Lu et al. 2024a), with the wtAAV virus production using HEK293 by co-infection with wtAAV2 and Ad5 (Case B) and by transfection of the wtAAV2 DNA-carrying pAV1 plasmid and the adenoviral helper gene-carrying pHelper plasmid (Case C) (Fig. 1).

**Fig. 2:**
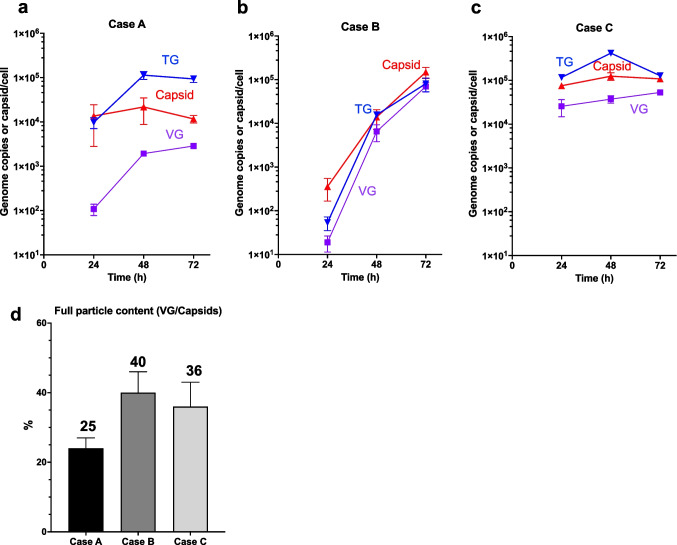
Production kinetics of viral components in three AAV systems. **a** GX2 (Case A), **b** wtAAV-Ad5 co-infection (Case B), and **c** wtAAV transfection (Case C). TG, the total intracellular virus genome; VG, Benzonase-resistant encapsidated vector genome. **d** Full particle content determined by dividing VG titer by capsid titer. The data are presented as the mean ± SD (*n* = 3)

The time profile of viral particle components, including capsids and viral genome in terms of both intracellular total genome (TG) and vector genome (VG, i.e., encapsidated genome), was rather different among the three cases (Fig. 2a-c). In Case C of wtAAV transfection, TG, capsids, and VG all reached high levels within 24 h. In Case A, TG and capsids also increased rapidly, but to a lesser extent than in Case C, while VG lagged behind TG and capsids. Compared to the other two cases, Case B of virus co-infection, even at the high MOI of both wtAAV and Ad5 used, the rise of TG, VG and capsid was much slower. The difference in the dynamics of these viral components is likely due to a key difference in the three systems. The transient transfection of Case C likely resulted in a very high initial intracellular viral genome and *rep*/*cap* gene copy number, leading to a very rapid increase in TG and capsids. GX2 has been characterized to have 22, 27, and 31 copies of Genome, Replication and Packaging Modules, respectively (Lu et al. 2024a). Upon induction of the multitude of copies of Rep68 and DBP caused the rapid increase of their gene expression as well as rapid replication of the viral genome. In Case B, the lower initial genome copy number of AAV delayed the rise of viral components, but eventually a very high level of virus was produced.

The VG titer at 72 h for both wtAAV cases (B and C) was more than ten times higher than the rAAV case of GX2. GX2 had a TG level comparable (~ 10^5^ copies per cell) to the two wtAAV cases, however, its capsid level was low (~ 10^4^ per cell) (Fig. 2a-c). GX2 produced a large excess of viral genomes, but not enough capsids to package them. But even with the high TG level, only about 30% of the capsid was packaged, accounting for less than 5% of the TG. In contrast, in both wtAAV systems, 40% or more of the TG was packaged in nearly 40% of the capsids (Fig. 2a-c). This difference in packaging efficiency is reflected in the full particle content as determined by dividing VG titer by capsid titer as seen in Fig. 2d. The lower full particle content in GX2 was confirmed by the assessment using Cryo-electron microscopy (Cryo-EM) (Supplemental Fig. S2a-c). The results strongly suggest that capsid production is a limiting factor for rAAV production in GX2 and that genome packaging was inefficient in GX2.

### AAV transcript and protein dynamics

**Fig. 3:**
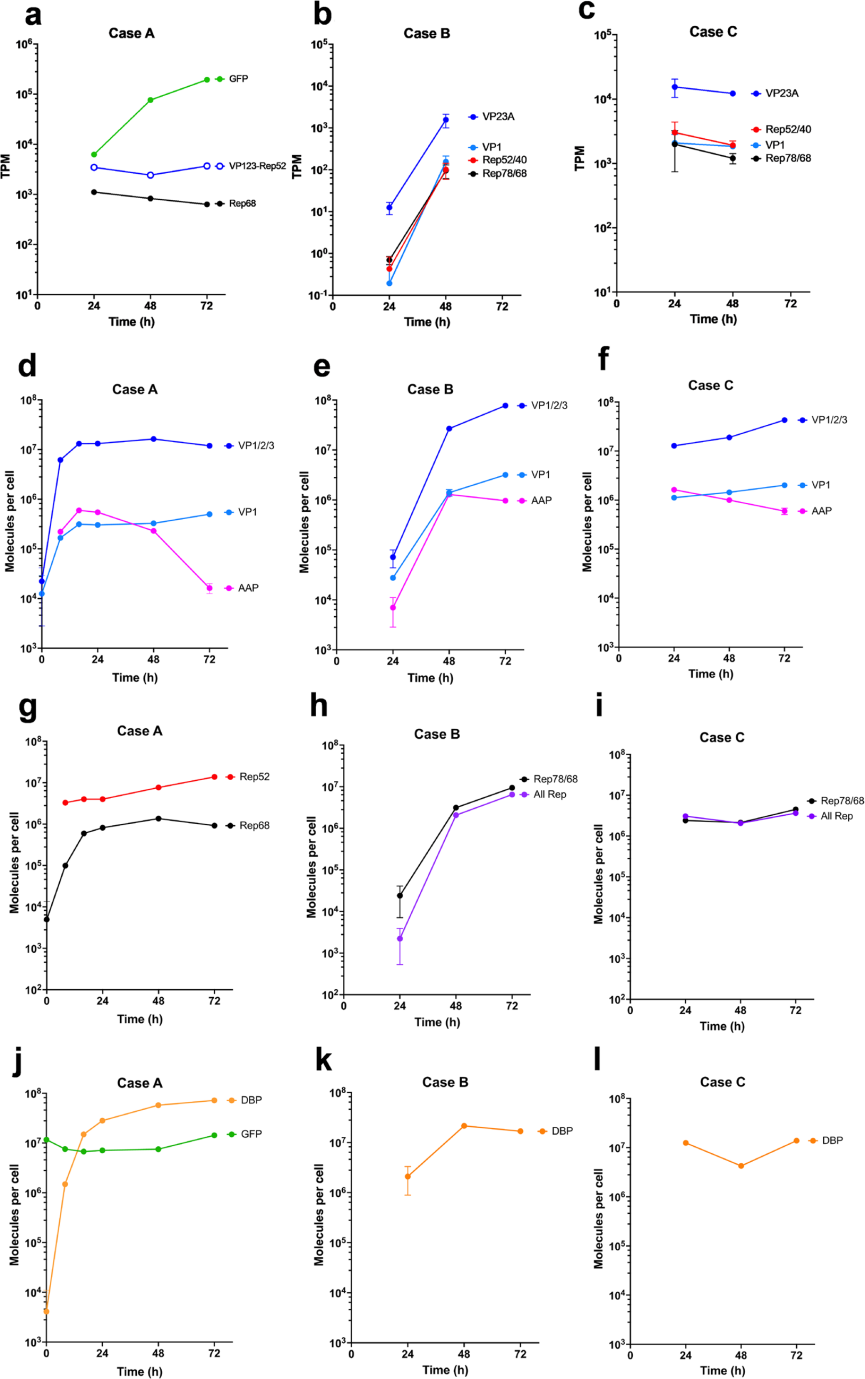
Time dynamics of viral transcripts and proteins in the production of AAV.** a**, **d**, **g**, **j** GX2 (Case A), **b**, **e**, **h**, **k** wtAAV-Ad5 co-infection (Case B), and **c**, **f**, **i**, **l** wtAAV transfection (Case C). VP123-Rep52 denotes the transcript encoding VP1/2/3 and Rep52 in GX2. VP23A denotes the truncated transcript encoding VP2, VP3, and AAP. The data are presented as the means ± SDs (*n* = 3)

The transcript and protein levels of various viral gene products in each AAV case were determined using RNA-seq and AQUA targeted quantitative proteomics, respectively. After 48 h of induction, the transcript of the cargo gene GFP in GX2 rapidly increased to 10^5^ levels of TPM and was the most abundant transcript of all cellular mRNA in GX2 (Fig. 3a). The intron-less AAV2 *cap* coding sequence (CDS) (designated as VP123) and the Rep52 CDS were transcribed into a single transcript of VP123-Rep52, which also increased rapidly in 24 h and stayed at this high level (Fig. 3a). Rep68, the lowest of all the transcripts transcribed from the three integrated modules, also reached high levels of > 10^3^ TPM (Fig. 3a and Supplemental Fig. S3b).

The two cases of wtAAV production had different viral transcript dynamics (Fig. 3b and c). In Case C, viral transcripts were at high levels by 24 h post transfection (hpt) and stayed at that level. In Case B, the viral transcript levels were much lower than Case C at 24 h and continued to rise rapidly until 48 h post infection (hpi). The transcription of both Cases B and C was driven by native viral promoters and regulatory machinery. The p5 and p19 promoters generate Rep78/68 and Rep52/40 transcripts, respectively. The entire transcript generated by the p40 promoter encodes VP1 (designated as VP1 transcript), while the truncated transcript encodes VP2, VP3 and AAP (designated as VP23A transcript). In these two wtAAV cases, Rep78/68 and Rep52/40 reached similar TPM level at either 24 or 48 h (Fig. 3b and c). VP23A was the most abundant of all viral transcripts at 48 h, while VP1 was about ten times less abundant. In both wtAAV cases, cell viability decreased quickly after 48 h and the RNA quality was not adequate for RNA-seq assay.

At the protein level, we saw a similar general kinetic behavior of viral proteins between Case A and C which is distinct from Case B, as seen in viral component transcript profile. In both Case A and C, viral proteins rose to high level by 24 h, while a slower rise was seen in Case B (Fig. 3d-l). At either 48 h or 72 h, VP1/2/3 (sum of VP1, VP2 and VP3) was more abundant than other measured viral proteins in the two wtAAV production cases (B and C), while DBP was more abundant than other measured viral proteins, including VP1/2/3, in GX2 (Case A) (Fig. 3d-l).

The VP1:VP2:VP3 ratio in the AAV capsid has been reported to be 1:1:10 (Gonçalves 2005). The proteome-measured relative abundance of VP1/2/3 to VP1 proteins for both wtAAV cases (B and C) was also approximately in the vicinity of 10 (Fig. 3e and f). This may reflect the regulation of the native promoters employed in both cases. The VP123 used as Packaging Module in GX2 has been engineered to tune the ratio of VP proteins (Lu et al. 2024a) and the measured VP1/2/3 and VP1 proteins level reflect that. Assuming that 60 VP subunits make up a capsid, it was estimated that after 72 h, 8%, 12%, and 13% of the VP proteins were assembled into the capsids in Case A, B, and C, respectively.

MAAP has been reported to be expressed from the + 1 frameshifted open reading frame in the VP1 region of the AAV cap gene, which may be in the spliced form of the p40 transcript that encodes VP2/3 and AAP (Elmore et al. 2021; Galibert et al. 2021). Thus, MAAP transcript was not quantified by RNA-seq. At the protein level, no high-confidence spectrum of a tryptic fragment of the MAAP protein was identified in the discovery stage of our proteomic tool development. Hence, MAAP was not quantified in the present study.

Regarding the Rep proteins, the two wtAAV cases express all large subunits (Rep78/68) and small subunits (Rep52/40), while GX2 produces only Rep68 and Rep52, with the latter co-transcribed with VP123. A faster early rise in Rep proteins and DBP was seen in Cases A and C similar to VP proteins. This early rise, in comparison with Case B, may contribute to the higher TG levels in Cases A and C at early time points. By subtracting the value of all Rep from Rep68 as an estimate of Rep52 protein level (Fig. 3g), one can see Rep68 protein was lower than Rep52 in GX2 at all time points. This is in contrast to both cases of wtAAV production, the curve of total Rep protein indicated that Rep52/40 may not be more abundant than Rep78/68 (Fig. 3h and i). We noted that for Cases B and C (Fig. 3h and i), the level of Rep78/68 was higher than that of all Rep proteins. It is possible that the level for all Rep proteins was underestimated. The target peptide for the segment which was present in all Rep proteins contained two glutamine residues which could be post-translationally deamidated. It is plausible that in Cases B and C deamidation of glutamine occurred, thus reducing the level of unmodified peptide.

Western blotting was performed to estimate the relative abundance of Rep proteins in the three cases, and Rep52 protein level was somewhat higher than Rep78/68 proteins in Cases B and C (Supplemental Fig. S4). This is consistent with the notion that total Rep was underestimated in Cases B and C. In the case of rAAV production, Rep52 was much more abundant than Rep68, whereas in the two wtAAV cases, Rep52/40 and Rep78/68 was present at similar levels (Supplemental Fig. S4). It can be seen that Rep52 protein was present at higher levels in GX2 than in the two wtAAV cases. Rep52 plays a key role in genome packaging of AAV (King et al. 2001). In spite of the higher level of Rep52, the full particle content seen in Case A was lower (Fig. 2d and Supplemental Fig. S2a-c). This suggests that Rep52/40 protein expression may not be the limiting factor for AAV genome packaging in GX2.

The abundance and dynamics of transcripts generated from adenoviral helper genes are shown in Supplemental Fig. S3. The schematic diagram of the alternatively spliced transcripts and protein coding sequences of *E1*, *E2,* and *E4* genes were shown in Supplemental Fig. S3a. The three AAV cases had different sets of adenoviral helper gene. Common to all three cases were *E1A* and *E1B* which are integrated in the host cell HEK293. Additionally, GX2 had inducible E4orf6 and DBP CDS integrated (Supplemental Fig. S1a). For Case B of wtAAV-Ad5 co-infection, Ad5 provided a complex set of helper functions, while in Case C of wtAAV transfection, adenoviral *E2A*, *E4,* and *VA RNA* were provided by the pHelper plasmids (Supplemental Fig. S1b).

### Representation of cargo transcripts and proteins in GX2

The relative abundance level of the transcript of different categories of genes in the three cases is shown in Supplemental Fig. S5. The cargo gene, i.e., *GFP* in Case A, was not only the most abundant viral transcript, but also the most abundant transcript of all cellular mRNA. *GFP* transcript in GX2, in spite of being under the control of lac operators and repressors, constituted nearly 8% of total cellular transcripts at 48 h and up to 20% at 72 h. At the protein level, GFP accumulated to 10^7^ molecules/cell level (Fig. 3j). Such excessive cargo gene expression is a waste of cellular resources. In Case B and C, the cargo gene is the *rep* and *cap* of wtAAV. Their *rep* and *cap* transcripts, under the control of the native viral promoters, constituted about 0.2% and 2.4% of total cellular mRNA at 48 h in Case B and C, respectively, and with more *cap* than *rep* transcripts.

It is noted that adenoviral helper gene transcripts including those of *E1A*/*E1B* in host cell and those from the gene modules and plasmids, were present to about 1% level at 48 h in all three cases in spite of the different ways those helper genes were encoded in or provided to the host cell (Supplemental Fig. S5). In Case B of wtAAV-Ad5 co-infection, other Ad5 transcripts constituted up to 6.4% of all cellular mRNA while wtAAV transcripts accounted for only 0.2%, indicating that Ad5 replication still dominated over AAV replication during co-infection. Although the transcripts of AAV genes constituted only a small fraction of total, it produced more encapsidated particles than in GX2. Co-infection is thus more efficient in the overall replication of AAV than the synthetic system GX2.

### Functional class enrichment at the transcript level in the three AAV cases

The transcriptome data were subjected to differential expression analysis using the 0 h of each case as the reference (uninduced for GX2, uninfected or untransfected for Cases B and C). The number of upregulated and downregulated DEGs for each pairwise comparison using a threshold of |Log_2_ FC|> 1 and FDR *p*-value < 0.05 is shown in Supplemental Fig. S6a-g. Cell viability was low and RNA quality was not good enough for RNA-seq after 72 h of virus co-infection or plasmid transfection, hence data at 48 h were used for functional enrichment analysis.

**Fig. 4:**
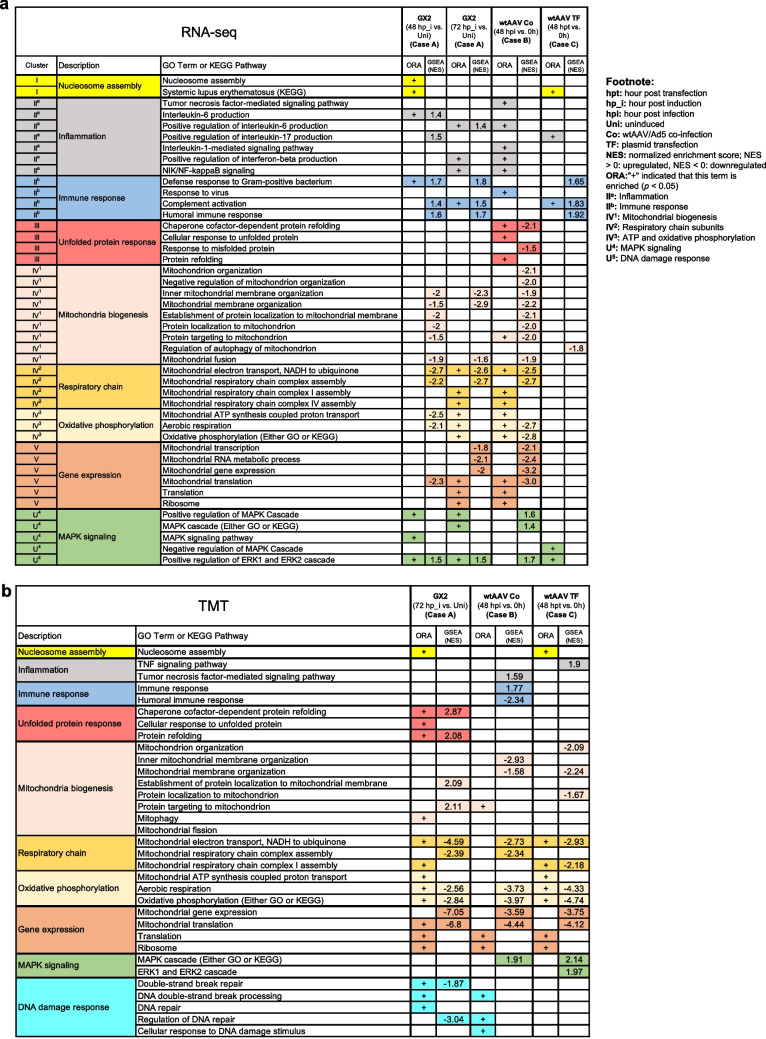
Functional classes enriched in the three AAV production systems at the transcript (**a**) and protein (**b**) levels. The enriched functional classes at the transcript and protein levels were identified using DAVID over-representative analysis and GSEA. For DAVID, a threshold of unadjusted *p*-value < 0.05 was used. A “ + ” sign indicates that the GO term or KEGG pathway was significantly enriched. For GSEA, the significance of enrichment of a functional class was based on the following criteria: NES > 1 or < -1, normalized *p*-value < 0.05 and *q*-value < 0.25

The DEGs identified in each case were subjected to overrepresentation analysis (ORA) by DAVID and GO and KEGG pathway databases. The enriched functional classes identified in each case were then clustered using DAVID. Five functionally relevant clusters are shown in Fig. 4a. Cluster I are related to nucleosome assembly, Cluster II consists of inflammation and immune response subgroups, Cluster III are mostly related to unfolded protein response, Cluster IV, mostly related to mitochondria, can be grouped into three sub-clusters, mitochondrial biogenesis (IV^1^), respiratory chain subunits (IV^2^) and adenosine triphosphate (ATP), and oxidative phosphorylation (IV^3^). A number of enriched GO terms, although not clustered together, are all involved in mitogen-activated protein kinase (MAPK) signaling and are also listed in unclustered (U).

Pre-ranked GSEA which uses expression of all transcripts as inputs as opposed to using only DEGs was also performed to identify enriched functional classes using GOBP database. GSEA uses a different method to assess functional class enrichment and complements DAVID. A consistent enrichment call by both DAVID and GSEA would lend credibility to the call. Indeed, many similar functional classes were identified as enriched by both DAVID and GSEA (Fig. 4a).

### Functional classes enriched at the protein level

To obtain a comprehensive overview of whole-cell protein expression, we used 16-plex TMT quantification to compare relative abundance of proteins at different time points. Overall, about 5000 proteins were identified in each case and 4,376 proteins were common in all three cases (Supplemental Fig. S7). A *p*-value < 0.05 threshold was adopted to make differentially expressed proteins (DEPs) calls on pairwise comparisons using the 0 h sample as the reference. 604 DEPs were identified in the comparison of uninduced GX2 and GX2 72 h (Supplemental Fig. S7a). For Cases B and C, about 400 proteins were differentially expressed 24 or 48 hpi (Supplemental Fig. S7b-d).

These DEPs were subjected to ORA using DAVID. The abundance ratio obtained from TMT quantification of all the identified proteins was also used as input to perform pre-ranked GSEA. Figure 4b tabulated the GO terms and pathways identified as enriched by DAVID and by GSEA. The list retained the same coloring for GO term or KEGG pathway as seen in Fig. 4a for easier visualization. The number of functional classes enriched was smaller than that obtained with transcriptome as the number of input genes was smaller.

### Comparison of functional class enrichment

As seen in Fig. 4a and b, many GO terms or pathways were called enriched by ORA or GSEA or both in more than one case. Looking at the cluster level, one can see most clusters in Cases A and B have commonly enriched terms at the transcript or protein level, or both. For example, Cases A and B each have several GO terms enriched in Cluster II^a^ at the transcript level (Fig. 4a). Some terms, such as positive regulation of interleukin-6 production, were commonly enriched, some others, such as interleukin-6 production, were enriched only in one case; however, it was noted that functions related to inflammation were enriched in Cases A and B. Interestingly, at the transcript level, Cases A and B have many common GO terms that were enriched in all functional clusters except Cluster I and III (Fig. 4a), and the number of common enriched GO terms was especially high in Cluster IV, V, and U, suggesting that at the transcript level, Cases A and B were more similar to each other than to Case C. At the protein level, Cases A and B again show a high degree of similarity in functional response. Cluster IV and V have many common enriched functional classes in all three cases, many of which were related to mitochondrial functions and activities (Fig. 4b). The results corroborated that seen in the transcriptome, but extends the observation to Case C.

The broad functional response at the transcript and protein level to rAAV/wtAAV production was summarized in Supplemental Fig. S8. All clusters were enriched at either transcript or protein level or both, except Cluster I, III and U^5^. The results support the notion that the production of virus/viral vector elicited similar functional responses.

### Host antiviral gene expression profiles

**Fig. 5:**
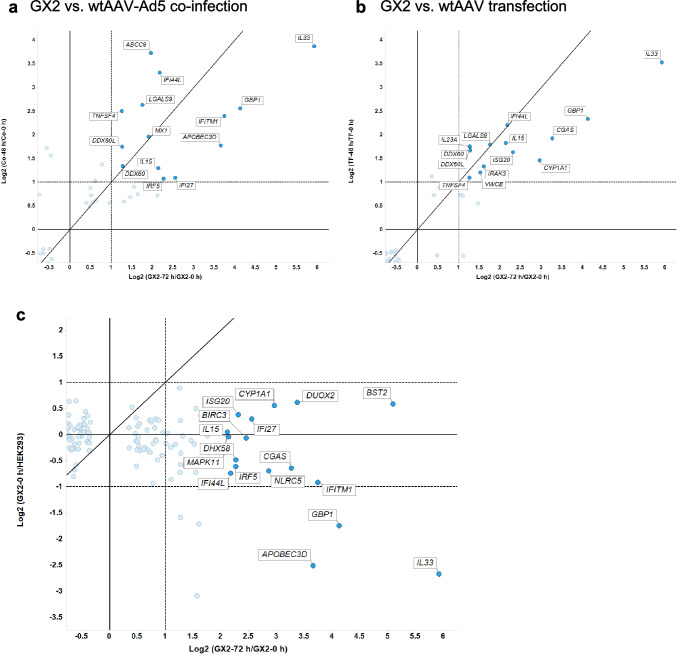
Fold change of the transcript of genes in the term Response to virus (GO:0009615) in three AAV cases. The fold change of antiviral genes upon production of wtAAV by co-infection (log_2_ (Co-48 hpi/Co-0 hpi)) (Case B) (**a**) and transfection (log_2_ (TF-48 hpt/TF-0 hpt) (Case C) (**b**) was compared to that by induction of GX2 (log_2_ (GX2-72 hpi/GX2-0 hpi) (Case A). In **c**, uninduced GX2 was compared to parental HEK293 cells (log_2_ (GX2-0 hpi/HEK293)). The dash line indicates a fold change of 2 (log_2_ FC = 1). All the gene symbol-labeled genes in **a** and **b** had an FDR *p*-value < 0.05 in both comparisons shown in the y- and x-axis. The labeled genes in (c) had FDR *p*-value < 0.05 in GX2-72 hpi to GX2-0 hpi comparison

Several innate immune system-related terms, such as interleukin, interferon, and tumor necrosis factor (TNF) production, were commonly enriched in all three cases as shown in Fig. 4a and b. In addition, we noted that the term Response to virus (GO:0009615) was enriched at the transcript level for Case B (wtAAV-Ad5 co-infection), but in Cases A and C, the number of DEG did not meet the enrichment criteria for this GO term. Nevertheless, many genes in this GO term were differentially expressed as the transcript level change in the three cases were shown in Fig. 5a and b. Several genes, including *IFI44L*, *DDX60*, *DDX60L*, *IFITM1*, and *ISG20*, were upregulated upon switching to rAAV or wtAAV production in at least two of the three cases. Interferon-induced protein 44 (IFI44) and interferon-induced protein 44-like (IFI44L) has been reported to interfere with respiratory syncytial virus (RSV) replication. Overexpression of *IFI44* or *IFI44L* was shown to restrict RSV infection at an early time point after infection, whereas knockout of these genes in mammalian airway epithelial cells resulted in increased levels of RSV titer (Busse et al. 2020). DDX60L (the DExD/H box helicase DEAD box polypeptide 60-like) is a restriction factor for hepatitis C virus (HCV) replication. Both *DDX60* and *DDX60L* are involved in the sensing of viral RNA. Knockout of these genes were shown to rescue HCV replication (Grünvogel et al. 2015).

Interferon-induced transmembrane protein 1 (*IFITM1*), an type I interferons (IFN)-responsive gene, encodes IFN-induced antiviral protein that inhibits entry of various viruses, including influenza A virus (IAV), HCV and severe acute respiratory syndrome coronaviruses (SARS-CoV) (Narayana et al. 2015; Shi et al. 2021). *ISG20* encodes interferon‐stimulated gene 20 kDa protein, which exhibits strong RNase capable of degrading viral and nonself RNAs. The protein encoded by *ISG20* was shown to inhibit a broad spectrum of viruses (Deymier et al. 2022). *IFI27*, which encodes interferon alpha inducible protein 27, was shown to counteract host-induced antiviral responses to RNA viral infection and therefore exhibit positive effect on IAV and SARS-CoV-2 replication (Villamayor et al. 2023). *IL33* and *GBP1* were differentially upregulated in all three cases but expressed in low levels. IL33 is a cytokine that interacts with IL1RL1/ST2 receptor which in turn activates NF-kappa-B and MAPK signaling pathways in target cells (Saravia et al. 2015). *GBP1* encodes a guanylate-binding protein, which was shown to target viral capsid protein to the lysosomal compartment (Glitscher et al. 2021).

Taken together, upregulation of these antiviral genes would have a negative effect on viral replication.

### Downregulation of mitochondrial biogenesis and metabolic genes

The enrichment of Cluster IV and V at both transcript and protein levels strongly suggests that mitochondrion functions related to mitochondrial translation, biogenesis, and oxidative phosphorylation were downregulated in all three cases. The fold change of all the genes in those terms was plotted in Supplemental Fig. S9 with the gene annotation shown in Supplemental Table S3. Approximately 70% of the downregulated mitochondrion-related DEGs were also shown to be mostly downregulated by TMT proteomics, giving credence to the downregulation of these mitochondrial activities in these cases.

As can be seen in Supplemental Fig. S9a, many genes in these terms were DEGs in both Cases A and B (log_2_ FC < -1), including *MRPL14*, *MRPL17*, *MRPL40*, *MRPL41*, *MT-ND6,* and *NDUFC2*, which were involved in mitochondrial translation (blue dots) or respiratory chain assembly (green dots). For Case C (Supplemental Fig. S9b), a general downregulated trend was seen, with most transcripts being slightly downregulated but not meeting the criteria for differential expression. This subtle downregulated trend in Case C was consistent with that seen at the protein level (Fig. 4b). Mitochondrial translation is key to the expression of mitochondrial genome-encoded genes including many proteins in the electron transport chain (Nolfi-Donegan et al. 2020), oxidative phosphorylation (Aibara et al. 2020) and is essential for mitochondrial biogenesis. Downregulation in both mitochondrial translation and energy metabolism likely indicates subdued energy metabolism in late stage of virus production. AAV genome replication, viral protein synthesis, and packaging are highly energy intensive processes. With the very high levels of genome amplification and viral protein synthesis seen in all three cases, the energy expenditure for viral replication is enormous. The diminished mitochondrial energy generation inevitably affects energy supply for AAV production.

Several GO terms in Cluster IV^1^ and V including Inner mitochondrial membrane organization (GO:0007007) and Mitochondrial gene expression (GO:0140053) were also involved in mitochondrial biogenesis and were enriched at the transcript or protein level in three cases (Fig. 4a and b). Downregulation of mitochondrial biogenesis was reported to cause increased mitochondrial stress and release of mitochondrial DNA (mtDNA) in some DNA and RNA virus infection (Sato et al. 2021). The released mtDNA was captured by cyclic guanosine monophosphate (GMP)- adenosine monophosphate (AMP) synthase (cGAS) and triggered host antiviral response. This mtDNA-activated cGAS pathway was proposed to play a role in innate control of certain viruses (Sato et al. 2021). Muhuri et al. (2021) have reported that rAAV-mediated stress may release mtDNA to activate the cytosolic DNA sensor cGAS. Upon DNA binding, cGAS promotes 2′3′-cyclic GMP-AMP (cGAMP) synthesis, which in turn activates the stimulator of interferon genes/interferon regulatory factor-3 (STING/IRF3) pathway and upregulates IFNs (Muhuri et al. 2021). It is possible that the changes we observed in mitochondrial gene expression at the transcript and protein levels are part of host cell antiviral response to counteract AAV infection.

### Absence of antiviral gene upregulation in uninduced GX2

A synthetic cell line-based rAAV production system bears a major difference from virus infection or plasmid transfection-based system. In the latter two cases, viral genes are introduced to the cell via infection or transfection, while in the synthetic system, the viral genes are integrated into the host cell genome and are always present. While these genes are not induced until production commences, it is plausible that leaky expression may occur and elicit host responses. We examined the expression level of antiviral genes by plotting the transcript fold change of uninduced GX2 and GX2 at 72 h after induction (Fig. 5c). As seen in Fig. 5c, compared to the parental HEK293 cells, none of the host viral response genes was upregulated before induction. This is in contrast to another synthetic cell line constructed using a similar approach to inducibly produce IAV in which many host antiviral genes were already upregulated in the synthetic cell line likely resulting from leaky expression of IAV viral genes (Phan et al. 2024).

### Upregulation of MAPK signaling during rAAV and wtAAV production

Several GO terms involved in MAPK signaling were commonly enriched and upregulated among three AAV cases (U^4^ in Fig. 4a and b) at both transcript and protein levels. The activation of MAPK signaling has been attributed to promote viral replication by increasing viral protein synthesis, viral genome replication, and suppressing host immune response and apoptosis (Kumar et al. 2018). As shown in Supplemental Fig. S10a and b, we further plotted and compared the DEGs in the term MAPK cascade (GO:0000165) among the three AAV cases. The DEGs that were commonly upregulated in all three AAV cases were summarized in Supplemental Table S4.

## Discussion

In this study, the process of rAAV production by the synthetic cell line GX2 was characterized at both transcript and protein levels and compared to wtAAV production by Ad5 co-infection and by plasmid transfection to gain insight into the viral and cellular kinetic features of AAV production. These production systems differ in many ways. In virus production systems, the viral genome was amplified by many orders of magnitude, resulting in very high expression of *rep* and *cap* transcripts and proteins. In contrast, in the rAAV production system, genome amplification merely amplifies the cargo gene, the gene copy number of *rep* and *cap* remained unchanged from what was integrated into the host cell genome. One hence anticipates that *rep* and *cap* expression would be substantially higher in Cases B and C than in Case A as was seen in the peak levels of Rep78/68 and VP1/2/3 proteins measured by AQUA (Fig. 3).

In the synthetic system, the expression of the adenoviral helper and *rep*, *cap* genes are under the control of the TetOn and CumateSwitch promoters, in contrast to the genes in Cases B and C, which are under the control of the native viral promoters. In the transfection-based production system (Case C), a large number of plasmids are loaded into the cell, wtAAV replication started with a very high copy number of genome template and viral genes, whereas in the virus co-infection system the event proceeded in a more natural way. These differences may contribute to the different kinetic behaviors in terms of gene expression and the assembly and packaging of viral particle in the three AAV production systems.

A recent study reported the transcriptome and proteome dynamics of rAAV-DJ production by triple plasmid transient transfection of HEK293 cells (Lu et al. 2024b). Although that study used a different AAV serotype, we nonetheless plotted the data from that case along with the three cases from this study as shown in Supplemental Fig. S11. The Rep78/68 and VP1/2/3 protein levels in both rAAV production systems were similar and lower than those in the wtAAV cases (Supplemental Fig. S11e-l). Although the Rep78/68 protein level was lower in the two rAAV cases (Supplemental Fig. S11i-l), the TG per cell in all four cases were not substantially different (Supplemental Fig. S11a-d). Furthermore, this is in spite of GX2 producing only Rep68 but not both Rep68 and Rep78 as in the other systems. This supports the notion that Rep68 alone is sufficient for efficient AAV genome amplification. The lower VP protein level in Case A may have contributed to its lower capsid titer than in the two wtAAV cases. Overall, only a relatively small fraction of VP protein subunits was assembled into capsids, approximately 12–13% for wtAAV systems and 8% for rAAV system. No capsid data was reported for the case of AAV-DJ because no suitable ELISA assay against serotype DJ was available. Assuming 60 VP1/2/3 molecule form a capsid the level of VP1/2/3 seen in the two rAAV cases would allow for a maximum of 2 × 10^5^ capsid/cell (i.e., at 100% efficiency of VP assembly). It is clear that the VP level in the two rAAV cases was too low to give a viral particle titer similar to that for wtAAV. An earlier report also suggested that capsid production was a limitation for rAAV production. Emmerling et al. improved rAAV yields with similar amounts of Rep protein by overexpression of VP (Emmerling et al. 2016). In addition to insufficient VP and capsid levels, a decline of AAP protein after reaching peak levels at around 24 h in GX2, which was not seen in the cases of AAV-DJ and wtAAV (Supplemental Fig. S11e-h), may have contributed to the lower productivity in GX2. AAP is a key player in AAV capsid assembly. Such a decline in AAV protein level was also seen in our previous study. Treatment with MG132, a proteasome inhibitor, reversed the decrease of VP1/2/3 and AAP proteins and increased capsid and VG level per cell by approximately tenfold (Lu et al. 2024a). Whether further increase in AAP level will increase the productivity even higher is yet to be examined.

We also noted that the efficiency of genome packaging in the two rAAV cases was low (~ 4–6% of AAV genome were packaged into encapsidated viral particle) as compared to wtAAV cases (~ 40–80%) (Supplemental Fig. S11a-d). Rep52 and Rep40 proteins play a key role in AAV genome encapsidation. However, since the level of Rep52 protein level was higher in Case A than in Case B and Case C, it is likely that the level of Rep52 is not the limiting factor for genome packaging. Packaging of AAV genome into capsids is affected by several factors, including ssDNA and capsid copy number, Rep52/40 proteins, host cellular DNA replication resources, such as deoxynucleotides, and DNA replication machinery (King et al. 2001; Weitzman and Fradet-Turcotte 2018; Sha et al. 2021). In addition, energy generated from ATP hydrolysis is required for Rep52/40 during genome packaging (King et al. 2001). Therefore, instead of overexpressing packaging protein Rep52/40, balance of Rep52/40, AAV genome, capsid, and host cellular resources may be important to improve genome packaging efficiency.

A comparison of the transcript distribution at 48 h reveals the high level of wasteful expression of the cargo gene *GFP* in both rAAV production systems (Supplemental Fig. S11m). Ideally, the cellular resources diverted to GFP synthesis should be redirected to *cap* protein synthesis in order to increase the productivity. We therefore postulate that boosting VP protein and capsid levels and suppressing cargo gene expression would have a positive effect on rAAV productivity. An additional increase in productivity may be achieved by modulating the gene expression dynamics in the synthetic cell GX2.

Interestingly, Case B of wtAAV/Ad5 co-infection produced a high level of encapsidated AAV particles compared to both rAAV production systems, in spite of the fact that it diverted much cellular resources to produce Ad5. At the transcript level, Ad5 transcripts constitutes about 6% of total cellular transcripts, while wtAAV transcript constitutes about 1% at 48 h. Ad5 provided a complex set of helper functions for AAV production. It is possible that those additional adenoviral factors help to create a suitable cellular environment that allows Case B to produce AAV more efficiently.

Virus replication requires cellular machinery and is dependent on cellular resources. Once infecting a host cell, virus interferes with or even shuts down some host cell functions, such as transcription and translation, and redirects these activities and resources toward the synthesis of viral components (Levene and Gaglia 2018). Conversely, as part of its defense mechanism, the host cell counters the infection by innate immune responses and inflammatory responses. For AAV, its replication is further dependent on the helper virus. In the case of rAAV production, the provided helper genes or helper virus also interacts with the host cells. Thus, host cellular responses during rAAV production may have profound effects on its productivity.

We investigated the host cellular response to wtAAV or rAAV production using the three different production systems at both transcript and protein levels. Among the three cases, Case B of wtAAV/Ad5 co-infection is closest to the “natural” process. At the transcript or protein level or both, co-infection elicited significant changes in functions of inflammation, innate immune response, unfolded protein response, and mitochondrial activities. Such host cell responses are commonly seen in different host cells in response to different viral infections (Grootjans et al. 2016; Mohanty et al. 2019). Granted that these host responses could be elicited by Ad5 infection alone with minimal contribution from wtAAV replication.

A similar pattern of host response was observed in Case A of the synthetic system GX2, indicating that host cells respond to the expression of AAV and helper genes and the production of AAV components in similar ways to that of wtAAV/Ad5 co-infected cells. This may reflect the similarity of GX2 cells, which contain multiple copies of the Genome Module, Replication Module and Packaging Module, to an Ad5 infected AAV latency model under uninduced conditions. Perhaps a bit unexpected about the host cell response was the lower degree of similarity in functional class enrichment when comparing Case C to Case B or A. Although Case B and Case C both involved wtAAV production, their similarity in functional enrichment was only apparent after including of both transcriptome and proteome analysis. Many factors may contribute to the lower similarity in functional enrichment for Case C. Case C differed from Case A and Case B in its use of polyethyleneimine (PEI) for transfection, which may induce different types of host cellular responses. Case C also used a high load of plasmids for transfection, resulting in very different dynamics of viral component production. Nevertheless, in all three cases, regardless of whether the genome was wtAAV or rAAV, whether the viral components were encoded by virus, plasmid vector, or integrated genetic modules, the host cell responded in a broadly similar manner, invoking pathways in immune response, inflammation, and MAPK signaling pathways. The earlier study on omic dynamics of rAAV-DJ transfection system also found that functional classes in inflammatory and immune response as well as MAPK signaling were enriched (Lu et al. 2024b).

A number of studies have used omics tools to probe host cell responses in rAAV production. An RNA-seq study of rAAV9 production by plasmid transfection also showed upregulated inflammatory response and type I interferon signaling pathway (Chung et al. 2023). MAPK signaling pathway was reported to be enriched at early time point. In a proteomic investigation of rAAV5 production by plasmid transfection of HEK293 cells, it was reported that the defense response was significantly enriched compared to untransfected cells (Strasser et al. 2021). Another transcriptomic study of rAAV2 production by plasmid transfection of HEK293 cells reported significant upregulation of the signaling pathways of pattern recognition receptors (PRR), including Toll-like receptor (TLR), retinoic acid-inducible gene-I (RIG-I)-like receptor (RLR), nucleotide oligomerization domain (NOD)-like receptor, and cytosolic DNA-sensing pathways (Wang et al. 2023).

Taken together, these omics studies showed that rAAV production elicited upregulation of inflammatory and innate immune responses as well as MAPK signaling in host cells, similar to the findings of this study, which spanned from rAAV to wtAAV. The inflammatory and innate immune responses triggered by viral infections have been reported to affect protein synthesis, cellular metabolism, and proliferation of host cells, which could suppress virus proliferation (Fritsch and Weichhart 2016). MAPK is an important signal transduction pathway known to be activated by a diverse group of viruses. The role of MAPK signaling in virus replication has been reviewed by Kumar et al. (2018). Viruses interact with members of the MAPK family — extracellular signal-regulated kinase (ERK), p38 and Janus kinase (JNK) — to manipulate cellular functions in their favor. Depending on the nature of the virus, MAPK signaling can either support or suppress viral replication (Rodriguez et al. 2014). The MAPK pathway could positively regulate the replication of a variety of viruses, including adenovirus, herpes simplex virus type 1 (HSV-1) and IAV (McLean and Bachenheimer 1999; O'Shea et al. 2005; Kumar et al. 2011). Modulation of MAPK signaling have the potential to affect AAV production.

We saw a general downregulation of mitochondrial activity and functions in Case A and B, as inferred from the transcriptome and proteome data. The mitochondrial downregulation was also seen in Case C but only at the protein level. Downregulation of the expression of genes encoding mitochondrial proteins is broadly seen in infection of DNA viruses, including modified vaccinia virus Ankara strain (MVA), or RNA viruses, including measles virus (MeV), RSV and IAV (Sato et al. 2021; Ye et al. 2021). Human cytomegalovirus (hCMV) transcribed a non-coding Beta2.7 RNA which interacts with mitochondrial respiratory chain complex I and inhibits host cell apoptosis and downregulates mitochondrial activity, thus enhancing viral replication (Combs et al. 2020). HCV has also been shown to disrupt host mitochondrial dynamics via promoting mitochondrial fission and subsequent mitophagy, which attenuates the apoptosis induced by HCV (Kim et al. 2014).

The present transcriptomic and proteomic study compared rAAV production using the synthetic cell GX2 with wtAAV production using two commonly used methods. We contend that wtAAV has been evolved in nature to develop an efficient virus production system, whereas our synthetic cell line was a laboratory design based on limited kinetic and stoichiometric insight into viral components. Compared to wtAAV production, the synthetic cell line produced insufficient amounts of VP proteins and capsids. While stoichiometrically there appeared to be a sufficient amount of genome copy to produce more encapsidated particles in GX2, the genome packaging efficiency was low. The kinetic behavior of genome amplification and capsid formation in GX2 differed from that in wtAAV co-infection, suggesting that optimizing the induction profile in GX2 and altering the kinetics of viral component synthesis may enhance the productivity and full particle content. Among the elicited host cell responses, several commonly observed functional classes related to antiviral responses were enriched in GX2 as well as wtAAV production systems. This suggests that suppressing antiviral responses in the synthetic cell system through cell engineering, as has been shown in some virus production systems, may enhance the productivity of rAAV (Hamamoto et al. 2013; Li et al. 2016).

## Supplementary Information

Below is the link to the electronic supplementary material.

Supplementary file1 (PDF 1783 KB)

## Data Availability

The data used to support the findings of this study are available from the corresponding author upon request. The RNA-seq data have been deposited in the NCBI database (BioProject: PRJNA1076915). The mass spectrometry proteomics data have been deposited to the ProteomeXchange Consortium via the PRIDE (Perez-Riverol et al. 2022) partner repository with the dataset identifier PXD050484.
