# Supplementary material for: Comparative transcriptomic and proteomic kinetic analysis of adeno-associated virus production systems
Source: Appl Microbiol Biotechnol. 2024 Jun 19;108:385. doi: 10.1007/s00253-024-13203-5 (PMC11186941; doi:10.1007/s00253-024-13203-5)
Supplement: Supplementary file 1 [file 253_2024_13203_MOESM1_ESM.pdf]

Supplemental Material

Applied Microbiology and Biotechnology

Comparative Transcriptomic and Proteomic Kinetic Analysis of Adeno-Associated Virus Production Systems

Yu-Chieh Lin, Min Lu, Wen Cai, Wei-Shou Hu*

Department of Chemical Engineering and Materials Science, University of Minnesota,

421 Washington Avenue S.E., Minneapolis, Minnesota 55455-0132

*Corresponding Author

Wei-Shou Hu

Address: 421 Washington Avenue SE

Minneapolis, MN 55455-0132 USA

Email: wshu@umn.edu

Phone: (612) 626-7630

Fax: (612) 626-7246

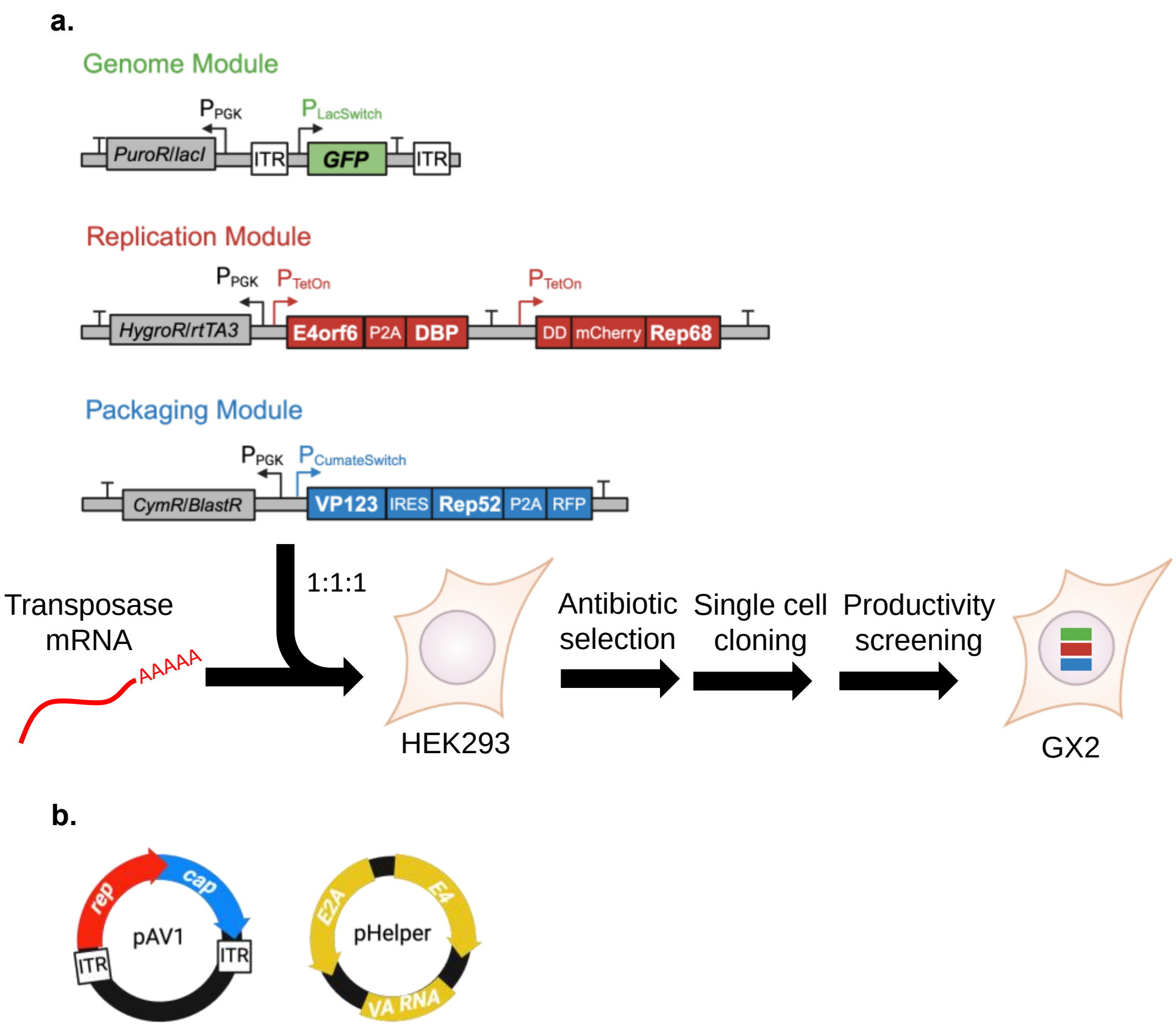

Supplemental Fig. S1. Schematic diagram of (a) gene modules used for the construction of rAAV2 producer cell line GX2 construction and (b) pAV1 and pHelper plasmids. (a) The genome of HEK293 cells were integrated with three transposon vectors: Genome Module contains a LacSwitch promoter-driven cargo gene *GFP* flanked by AAV2 inverted terminal repeats (ITRs) with a repressor gene *lacI* linked to a puromycin resistance gene driven by a phosphoglycerate kinase promoter (P_{PGK}). Replication Module contains an adenoviral helper E4orf6 and a DNA binding protein (DBP) CDS, and a destabilization domain (DD) and mCherry-tagged AAV2 Rep68 CDS under the control of inducible TetOn promoters with a reverse Tet transactivator gene *rtTA3* linked to a hygromycin resistance gene driven by a P_{PGK}. Packaging Modules contains an inducible CumateSwitch promoter-driven intron-less AAV2 *cap* CDS (VP123) linked to a Rep52 CDS via an internal ribosome entry site (IRES). In addition, the Rep52 CDS is linked through P2A to a small ultra-red fluorescent protein (smURFP) CDS. This module also contains a Cym repressor gene *CymR* linked to a blasticidin resistance gene driven by a P_{PGK}. Followed by antibiotic selection, single-cell cloning and productivity screening, clone GX2 was obtained. (b) pAV1 plasmid contains wtAAV2 genome, while pHelper plasmid contains adenoviral helper genes *E2A*, *E4* and *VA RNA*.

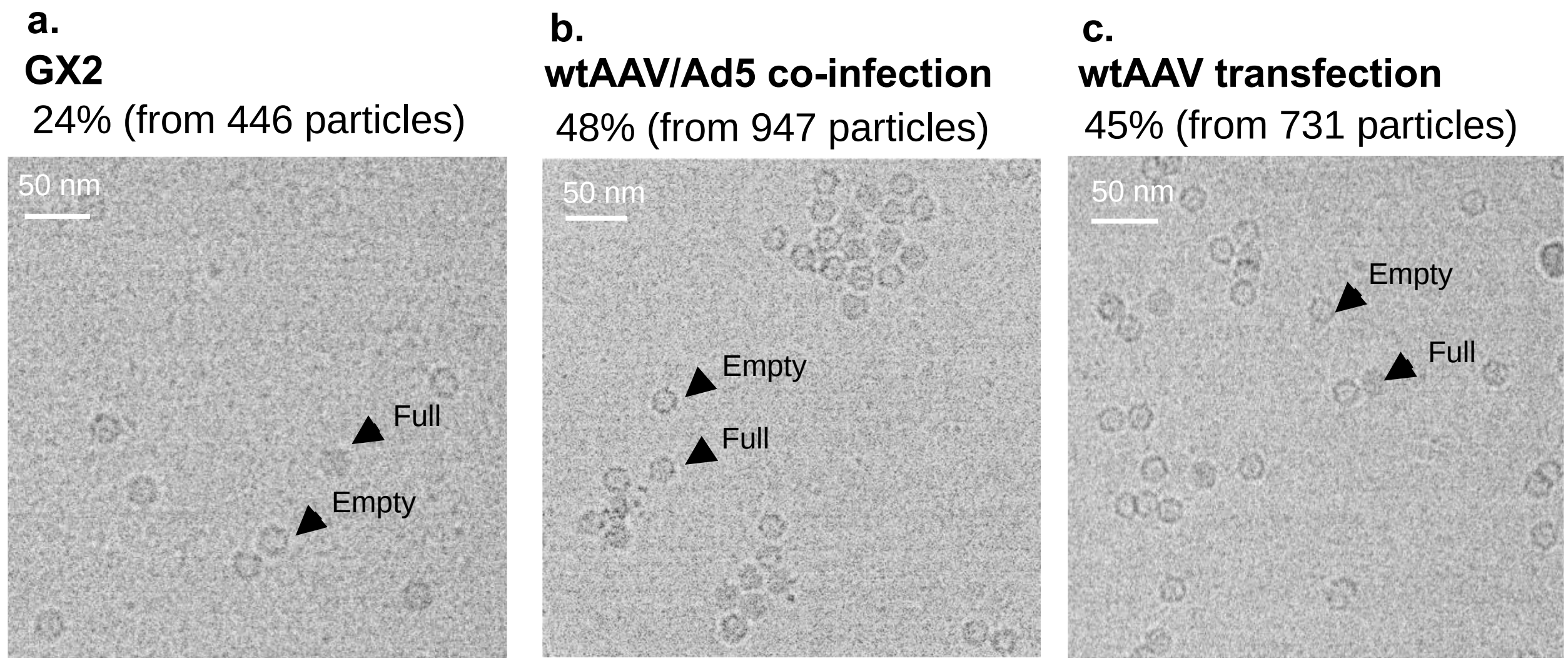

Supplemental Fig. S2. Electron microscopic determination of full particle contents of viral particles produced in Case A (GX2) (a), Case B (wtAAV-Ad5 co-infection) (b) and Case C (wtAAV transfection) (c). AAV particles were produced by induced GX2, wtAAV-Ad5-infected or pAV1-pHelper-transfected HEK293 cells in multiple 150 mm cell culture dishes and followed by purification using affinity chromatography. The procedure of AAV purification, sample preparation and equipment for cryogenic electron microscopy (Cryo-EM) were as described (Lu et al. 2024). The captured Cryo-EM images underwent image processing and the full and empty were manually counted to determine the full particle contents.

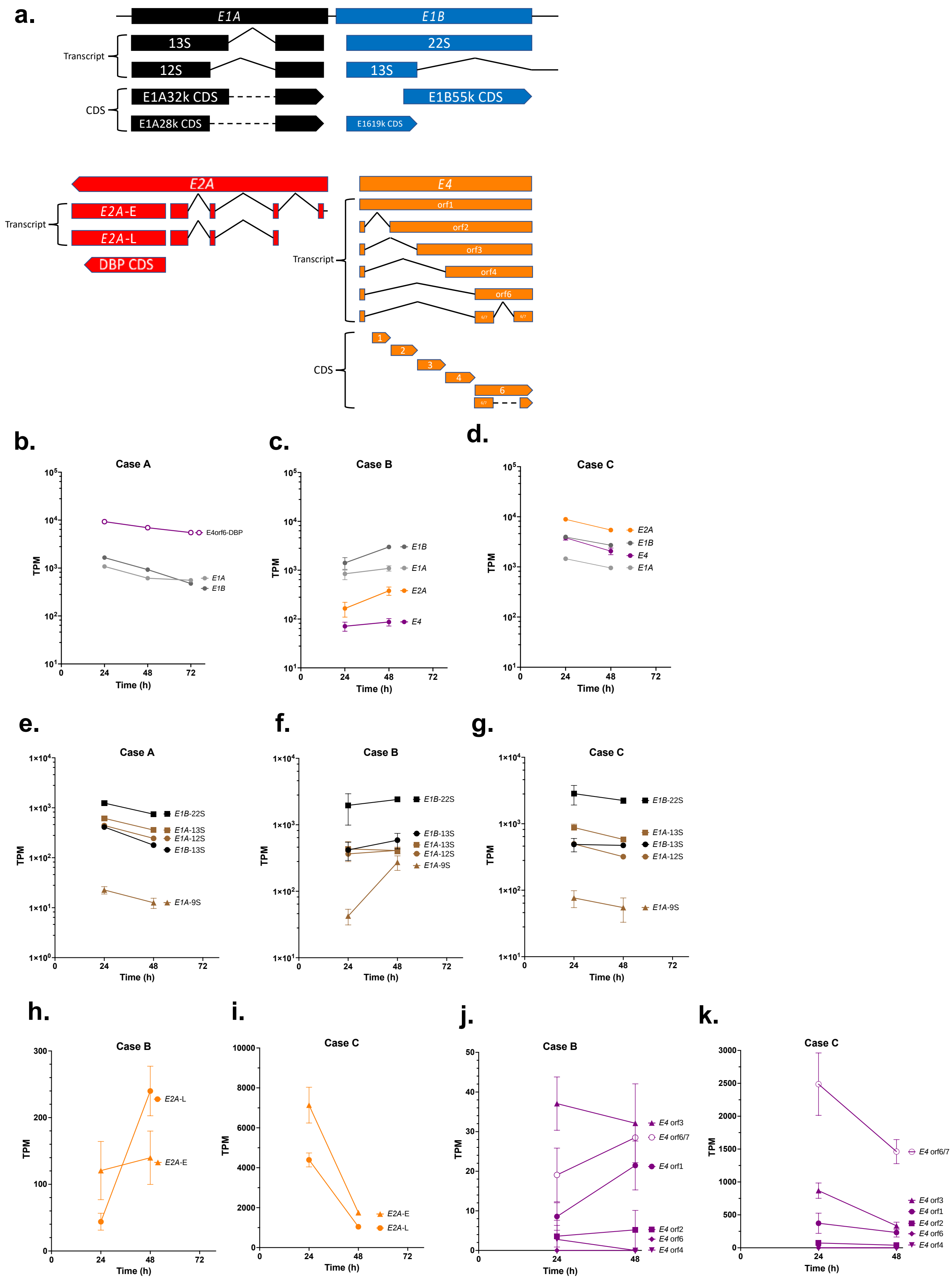

a.
E1A
E1B
Transcript
13S
12S
22S
13S
CDS
E1A32k CDS
E1A28k CDS
E1B55k CDS
E1619k CDS
E2A
E4
E2A-E
E2A-L
DBP CDS
orf1
orf2
orf3
orf4
orf6
CDS
b.
Case A
c.
Case B
d.
Case C
e.
Case A
f.
Case B
g.
Case C
h.
Case B
i.
Case C
j.
Case B
k.
Case C
TPM
Time (h)
E4orf6-DBP
E1A
E1B
E2A
E4
E1B-22S
E1A-13S
E1A-12S
E1B-13S
E1A-9S
E2A-L
E2A-E
E4 orf3
E4 orf6/7
E4 orf1
E4 orf2
E4 orf6
E4 orf4

Supplemental Fig. S3. Transcripts of adenoviral helper *E1A*, *E1B*, *E2A* and *E4* genes and their time dynamic during AAV production in three production systems. (a) The schematic diagram of the alternatively spliced transcripts and protein coding sequences of *E1*, *E2* and *E4* genes. (b, e): GX2 (Case A); (c, f, h, j): wtAAV-Ad5 co-infection (Case B); and (d, g, i, k): wtAAV transfection (Case C). E4orf6-DBP denotes the transcript encoding E4orf6 and DBP in GX2. The data are presented as the means±SDs (n=3). As shown in (b-d), *E1A* and *E1B* transcripts were expressed at high levels in HEK293. A TPM level of about 10^3 was seen at 24 h in all three cases. Ad5 co-infection provided additional *E1A* and *E1B* gene copies in Case B, but no major further increase of their transcripts was seen compared to other two cases. *E1B* transcript was more abundant than *E1A* transcript in all three cases. The abundance and dynamics of different alternatively spliced transcripts generated from *E1A* and *E1B* gene as shown in (e-g). The abundance level of different transcripts was in the same range among the three cases, but the dynamics varied somewhat in three cases. *E1B*-22S, encoding E1B-55k protein, was the most abundant among all alternatively spliced transcript isoforms of *E1A* and *E1B* in all three cases. The E1B-55k protein plays a key role in regulation of host cell response, which blocks p53 from inhibiting the cell cycle and prevents p53 from inducing apoptosis (Debbas and White 1993). Adenoviral *E2A* and *E4* are two additional helper genes commonly used in AAV production. They are both provided through co-infected Ad5 in Case B, and through the pHelper plasmids in Case C. In GX2, only E4orf6 CDS in *E4* and DBP CDS in *E2A* were provided as a single transcript E4orf6-DBP. In GX2, E4orf6-DBP transcript reached 10^4 transcript per million (TPM) levels at 24 h and stayed at this high level as shown in (b). In Case C, *E2A* and *E4* were also at a very high level as E4orf6-DBP transcript in GX2 as shown in (b) and (d). Surprisingly, the transcript expression of *E2A* and *E4* was more moderate in Case B as shown in (c). At 48 h, TPM of both *E2A* and *E4* were an order of magnitude lower than those in Case C. The lower *E2A* expression at the transcript level was also reflected at the protein level. At 24 h, DBP in Case B had a lower abundance than in Case A and C (Fig. 3j-l). The adenoviral *E2* and *E4* genes in either Ad5 or pHelper plasmids have alternatively spliced transcripts of varying abundance, in contrast to GX2, which has only DBP and E4orf6 CDS integrated, as shown in (h-k). Notably, transcripts encoding E4orf6/7, E4orf3 and E4orf1 had relatively higher expression levels compared to other *E4* alternatively spliced transcripts in either Case B and C. As reported, E4orf6/7 interacts with E2F transcription factors and plays a role in the regulation of DNA replication and cell cycle progression (Schaley et al. 2005). E4orf3 is involved in supporting viral replication and the inactivating cellular antiviral defenses (Araujo et al. 2005). E4orf1 encodes a protein that enhances host cell glucose uptake (Shastri et al. 2018). Taken together, it is

likely that these *E4*-encoded proteins could provide additional helper functions to both wtAAV cases and enhance their AAV productivity.

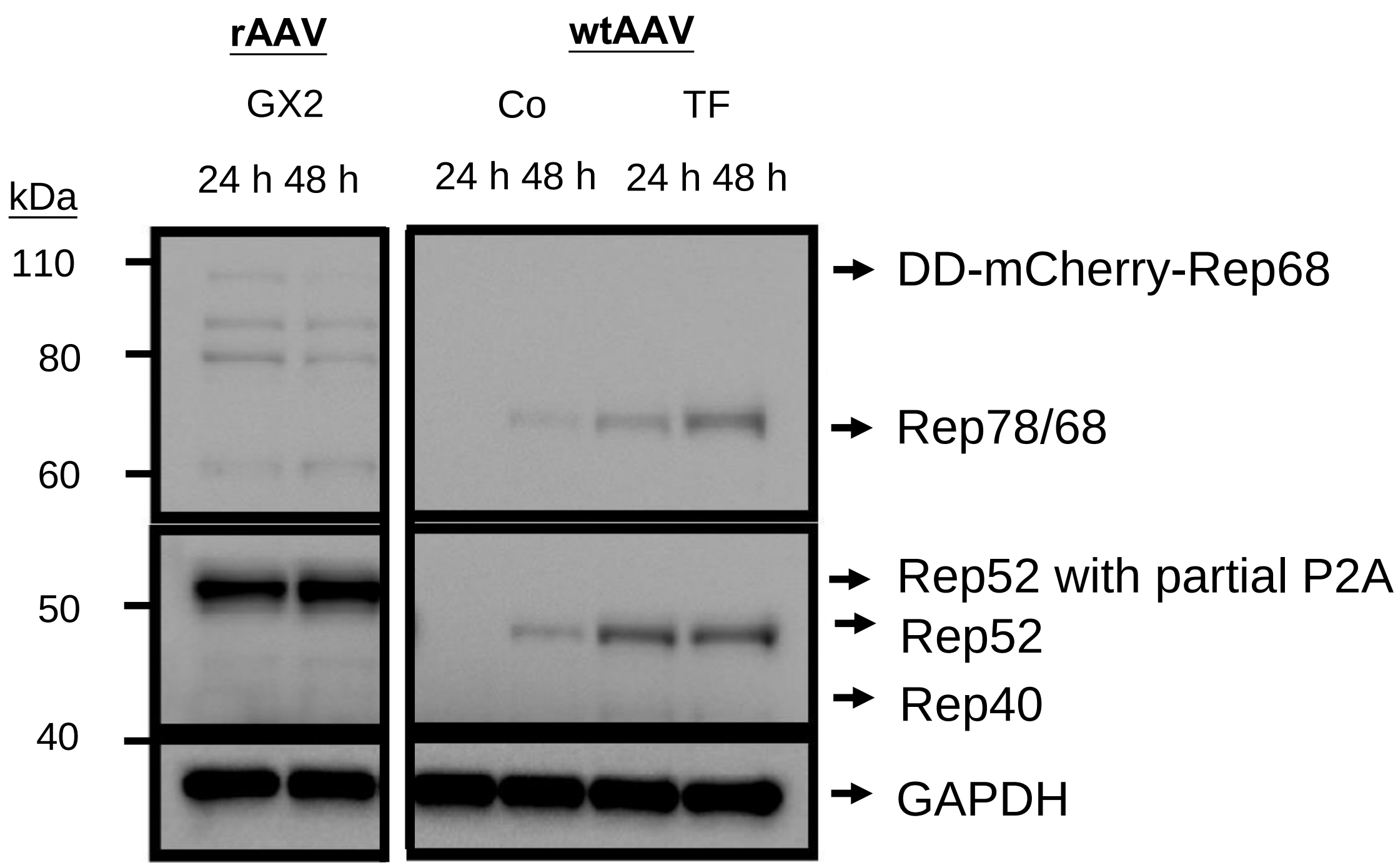

Supplemental Fig. S4. Western blot analysis of Rep protein. Samples were harvested at 24 and 48 h post induction, co-infection or transfection. The harvested cells were lysed using NP-40 cell lysis buffer containing protease inhibitors (Roche, Indianapolis, IN, USA). The obtained cell lysates were subsequently clarified via centrifugation and protein concentration was determined by bicinchoninic acid (BCA) protein assay (Thermo Fisher Scientific, Waltham, MA, USA). 10 μg of denatured and reduced proteins was loaded to each lane of NuPAGE Bis-Tris gels (Thermo Fisher Scientific, Waltham, MA, USA) and subsequently transferred to the 0.45 μm nitrocellulose membrane (Bio-Rad, Hercules, CA, USA). The blotted membranes were then incubated with mouse anti-AAV Rep protein (Fitzgerald, Gardner, MA, USA) and anti-GAPDH monoclonal antibodies (mAbs) (Cell Signaling Technology, Danvers, MA, USA) and corresponding anti-mouse IgG conjugated with alkaline phosphatase mAb (Sigma-Aldrich, St. Louis, MO, USA). The chemiluminescent signal was detected using the Immun-StarTM AP Chemiluminescence Kits (Bio-Rad, Hercules, CA, USA). Case A: GX2; Case B: wtAAV-Ad5 co-infection; Case C: wtAAV transfection. AAV2 Rep68 protein in GX2 was tagged by a destabilization domain (DD) and mCherry. The DD-tagged protein was ubiquitinated and degraded and seen in multiple bands (80-110 kDa). AAV2 Rep52 protein in GX2 was linked with small ultra-red fluorescent protein (smURFP) by the self-cleaving peptide P2A, a 22 amino acid peptide. After cleavage, a segment of 21 extra amino acids from P2A was attached to the Rep52 protein. Therefore, bands of Rep52 with partial P2A were observed in the Western blotting membrane.

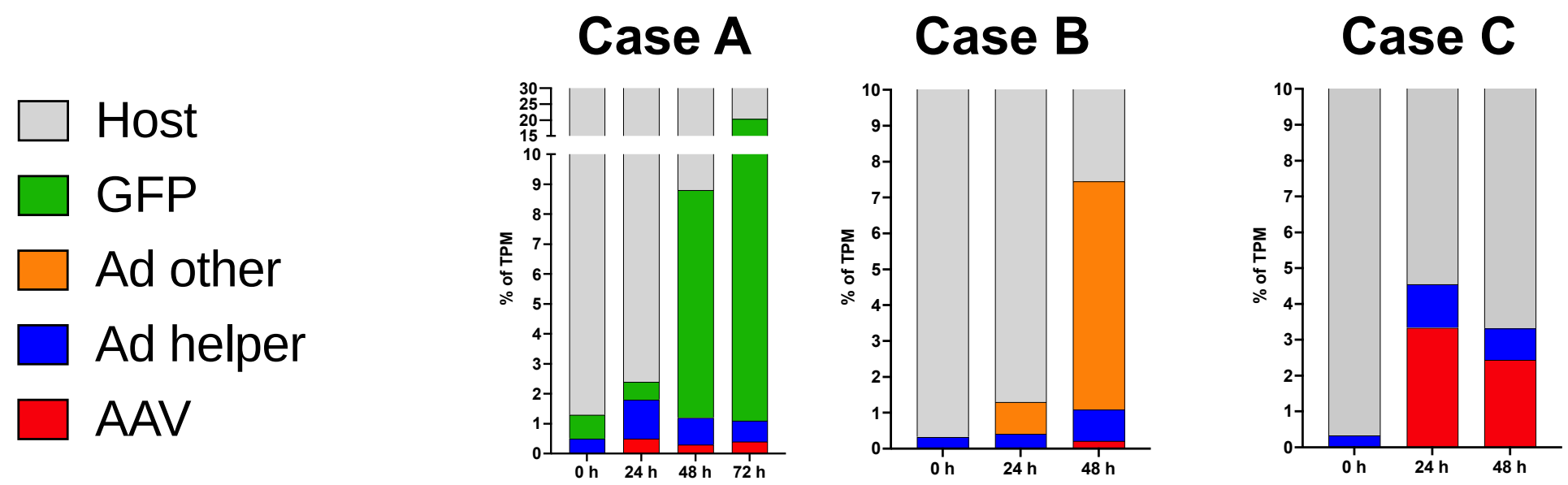

Transcript	Time (h)	GX2 (Case A)	wtAAV-Ad5 co-infection (Case B)	wtAAV transfection (Case C)
GFP	0 h	**0.3**±0.01	**N/A**	**N/A**
	24 h	**0.6**±0.01	**N/A**	**N/A**
	48 h	**7.6**±0.01	**N/A**	**N/A**
	72 h	**19.3**±0.8	**N/A**	**N/A**
AAV genes	0 h	**0**	**0**	**0**
	24 h	**0.5**±0.01	**0.002**±0.00	**3.3**±1.2
	48 h	**0.3**±0.00	**0.2**±0.07	**2.4**±0.2
	72 h	**0.4**±0.01	**N/A**	**N/A**
Ad helper genes	0 h	**0.5**±0.01	**0.3**±0.1	**0.3**±0.1
	24 h	**1.3**±0.01	**0.4**±0.1	**1.9**±0.2
	48 h	**0.9**±0.01	**0.9**±0.08	**0.9**±0.2
	72 h	**0.7**±0.01	**N/A**	**N/A**
Ad other genes	0 h	**N/A**	**0**	**N/A**
	24 h	**N/A**	**0.9**±0.6	**N/A**
	48 h	**N/A**	**6.4**±1.9	**N/A**
	72 h	**N/A**	**N/A**	**N/A**
Host cell genes	0 h	**99.2**±0.02	**99.7**±0.1	**99.2**±0.02
	24 h	**97.7**±0.03	**98.7**±0.4	**93.2**±2.5
	48 h	**91.1**±0.01	**92.6**±2.0	**96.2**±2.4
	72 h	**79.6**±0.8	**N/A**	**N/A**

Supplemental Fig. S5. Percentage of transcripts of different categories of genes in the three AAV systems. In the upper figures, different colors indicate different categories of genes, and the y-axis indicates the percentage (%) of total transcripts per million (TPM). "Ad other" represents the transcripts generated by all adenoviral genes except helper genes. The details of the percentage of TPM of each category of genes in the three AAV cases at each measured time point are listed in the table below. N/A: Not applicable.

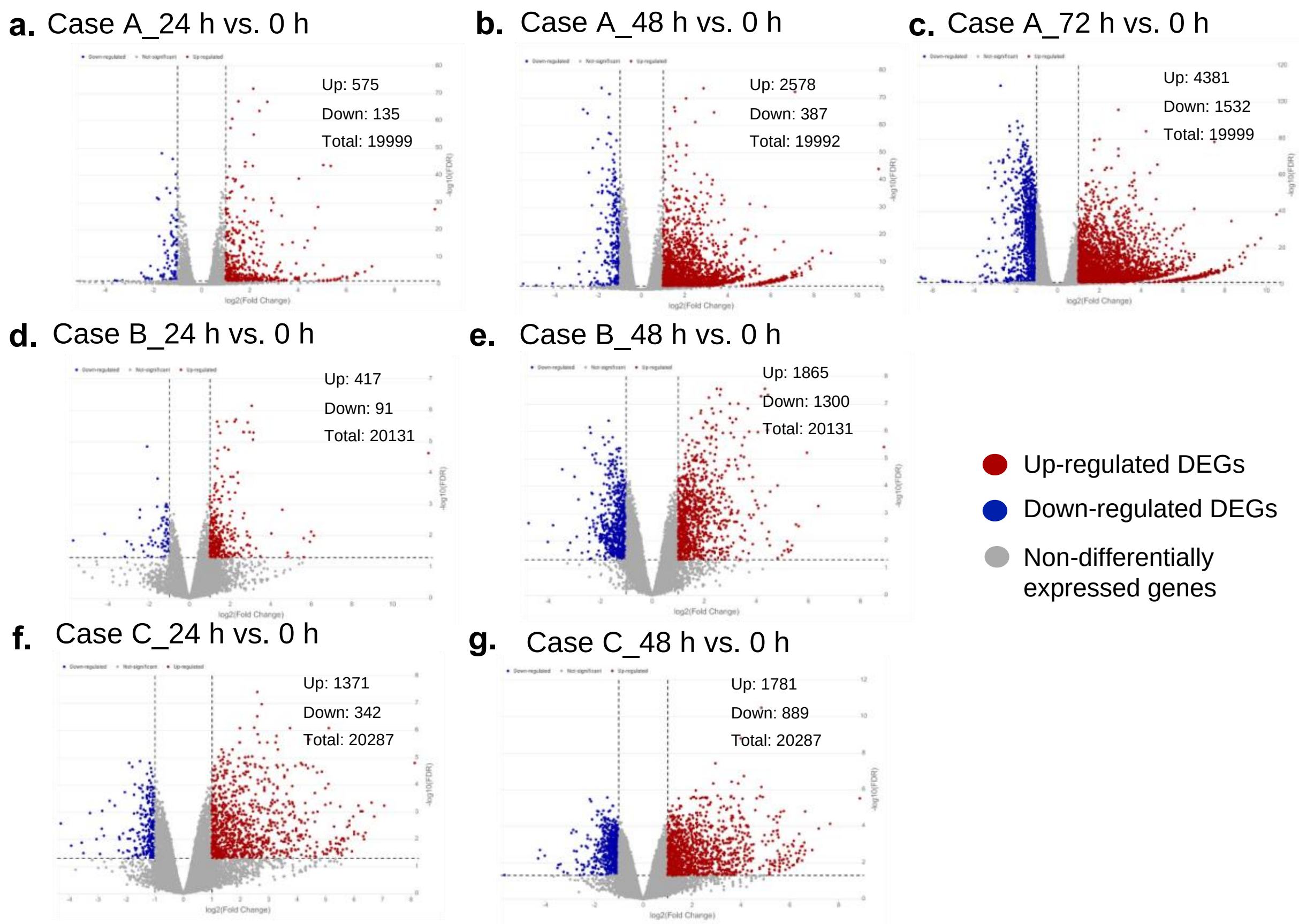

Supplemental Fig. S6. Differential expression of transcripts in three AAV production systems. (a-c): rAAV production by synthetic cell line GX2 cells induced with 10 µg/mL doxycycline and 90 µg/mL cumate (10D90C)) for 24, 48 or 72 h were compared to that of uninduced GX2 cells (0 h); (d, e): wtAAV production by co-infection of HEK293 cells with wtAAV and Ad5. 24, 48 hours post infection (hpi) samples were compared to that of uninfected cells (0 hpi); (f, g): wtAAV production by plasmid transfection of HEK293 cells. Samples of 24, 48 hours post transfection (hpt) were compared to that of un-transfected cells (0 hpt). For all the comparisons, a threshold of $|\text{Log}_2$ fold change (FC)$|$ > 1 and FDR *p*-value < 0.05 was used to identify differentially expressed genes (DEGs). In all three cases, about 20,000 transcripts were identified of which about 2600 to 3000 were differentially expressed at 48 h post induction, co-infection or transfection. The peak number of DEGs was seen at 72 h for Case A as shown in (c).

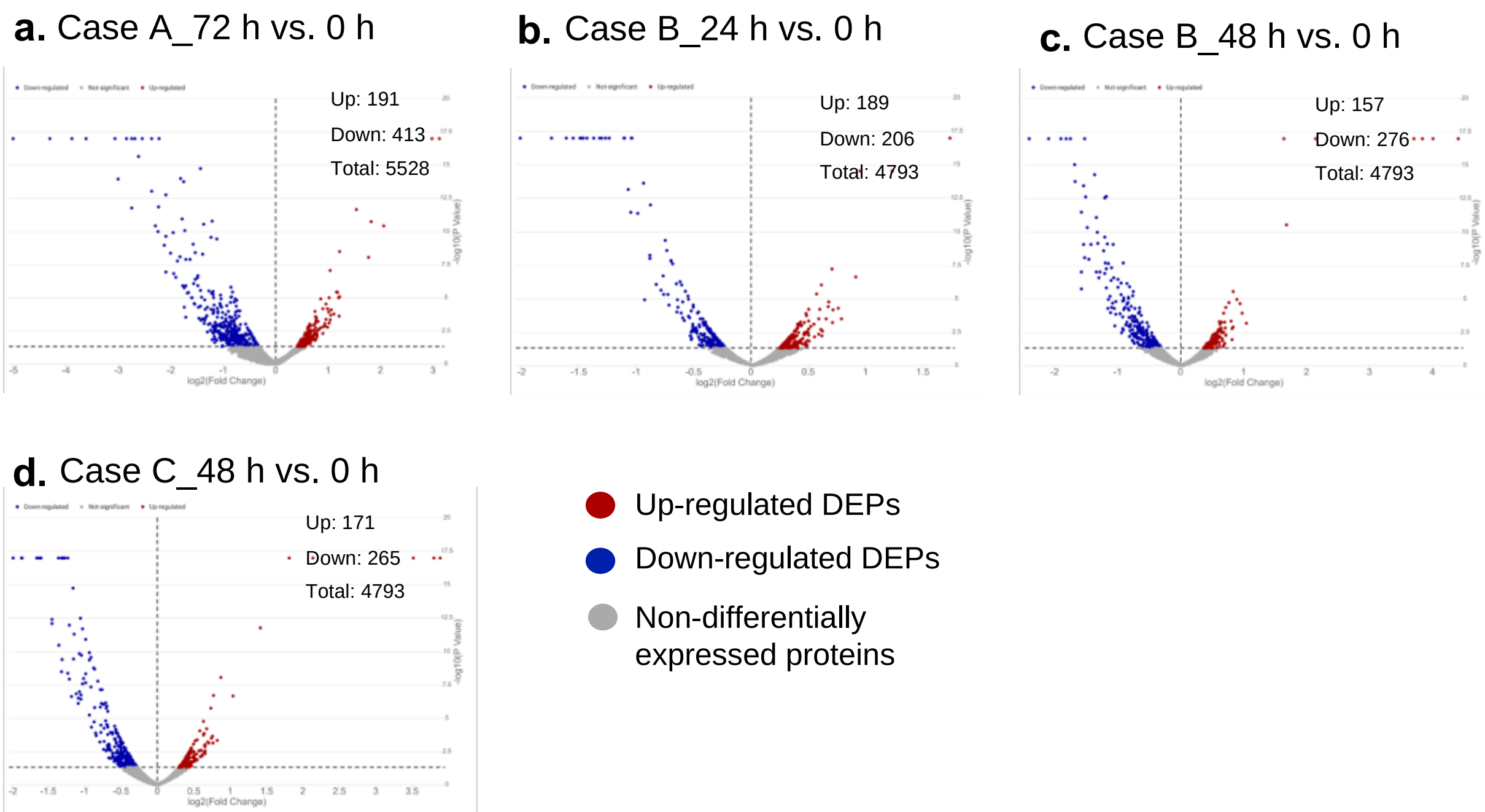

Supplemental Fig. S7. Differential expression of proteins upon switching to different AAV production cases. (a) For Case A of rAAV production, the proteome of 10D90C-induced GX2 cells (72 h) was compared to that of uninduced cells (0 h). (b, c) For Case B of wtAAV production, the proteome of wtAAV-Ad5 co-infected HEK293 cells (24, 48 hpi) was compared to that of uninfected cells (0 hpi). (d) For Case C of wtAAV production, the proteome of pAV1-pHelper co-transfected HEK293 cells (48 hpt) was compared to that of un-transfected cells (0 hpt). For all the comparisons, a threshold of unadjusted *p*-value < 0.05 was used to identify differentially expressed proteins (DEPs). In all three cases, about 5,000 proteins were identified. In each comparison, about 400 to 500 proteins were differentially expressed.

Functional Annotation Cluster	GX2 **(Case A)**		wtAAV-Ad5 co-infection **(Case B)**		wtAAV transfection **(Case C)**	
	RNAseq	TMT	RNAseq	TMT	RNAseq	TMT
Nucleosome assembly (I)	+	+			+	+
Inflammation (II[a])	+		+	+	+	+
Immune response (II[b])	+		+	+	+	+
Unfolded protein response (III)		+	+			
Mitochondria biogenesis (IV[1])	+	+	+	+	+	+
Respiratory chain (IV[2])	+	+	+	+		+
Oxidative phosphorylation (IV[3])	+	+	+	+		+
Gene expression (V)	+	+	+	+		+
MAPK signaling (U[4])	+		+	+	+	+
DNA damage response (U[5])		+		+		

Supplemental Fig. S8. Overview of functional clusters enriched at transcript and protein levels in three AAV systems. A "+" sign indicates that at least one GO term/KEGG pathway was enriched in a particular cluster at the transcript or protein level.

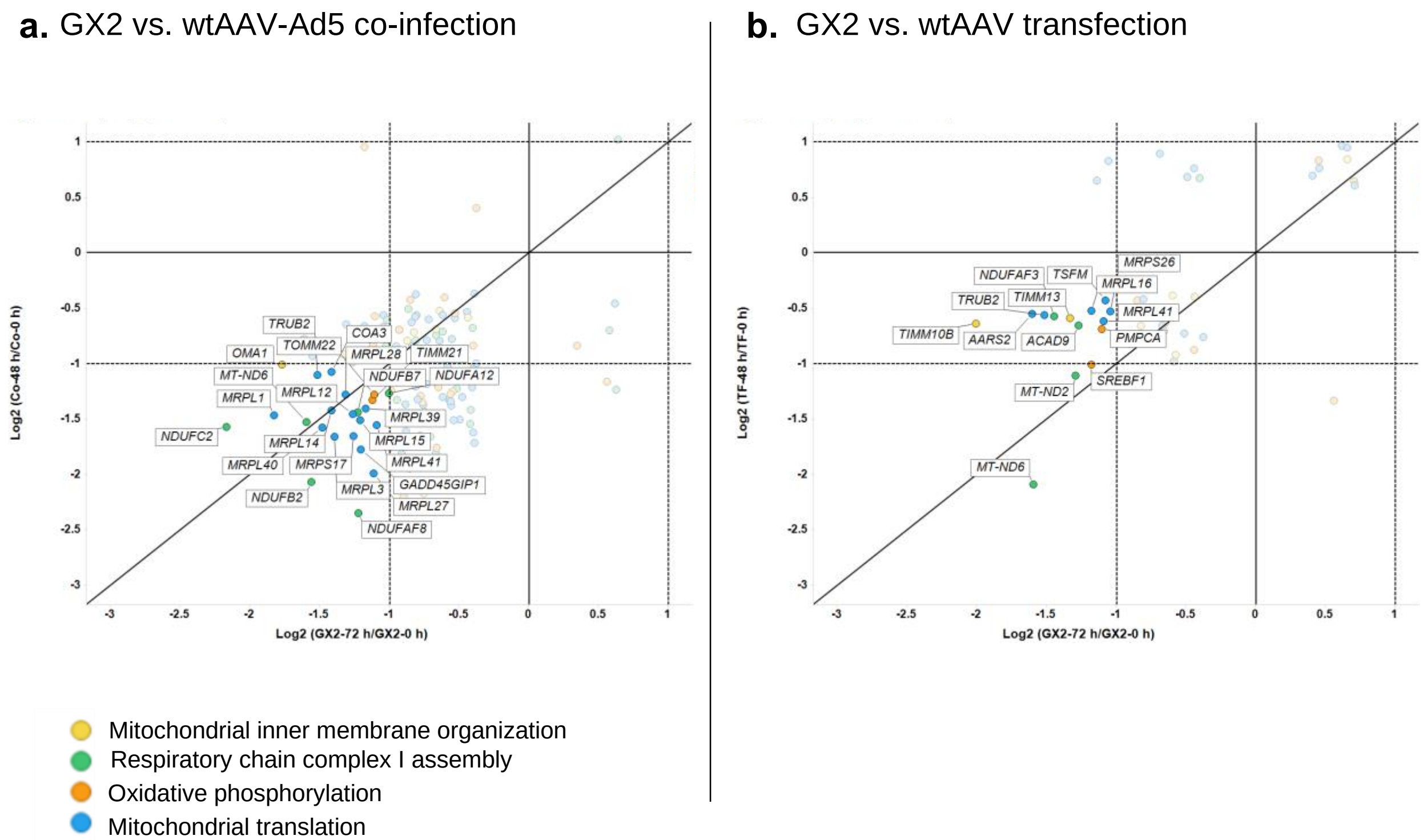

Supplemental Fig. S9. Transcript fold change of mitochondrion-related genes in three AAV production systems. The fold change of all the genes in the term Inner mitochondrial membrane organization (GO: 0007007), Mitochondrial respiratory chain complex I assembly (GO:0032981), Oxidative phosphorylation (GO:0006119) and Mitochondrial translation (GO:0032543) were plotted. The fold change upon production of wtAAV by co-infection ($\log_2$ (Co-48 hpi/Co-0 hpi)) (Case B) (a) and transfection ($\log_2$ (TF-48 hpi/TF-0 hpi) (Case C) (b) was compared to that by induction of GX2 ($\log_2$ (GX2-72 hpi/GX2-0 hpi). The dash line in (a) and (b) mark downregulation by twofold ($\log_2$ FC = -1). All labeled mitochondrion-related genes had an FDR *p*-value below 0.05 in each comparison shown in the y-axis and x-axis titles of (a) and (b). Different colors indicate genes in different GO terms.

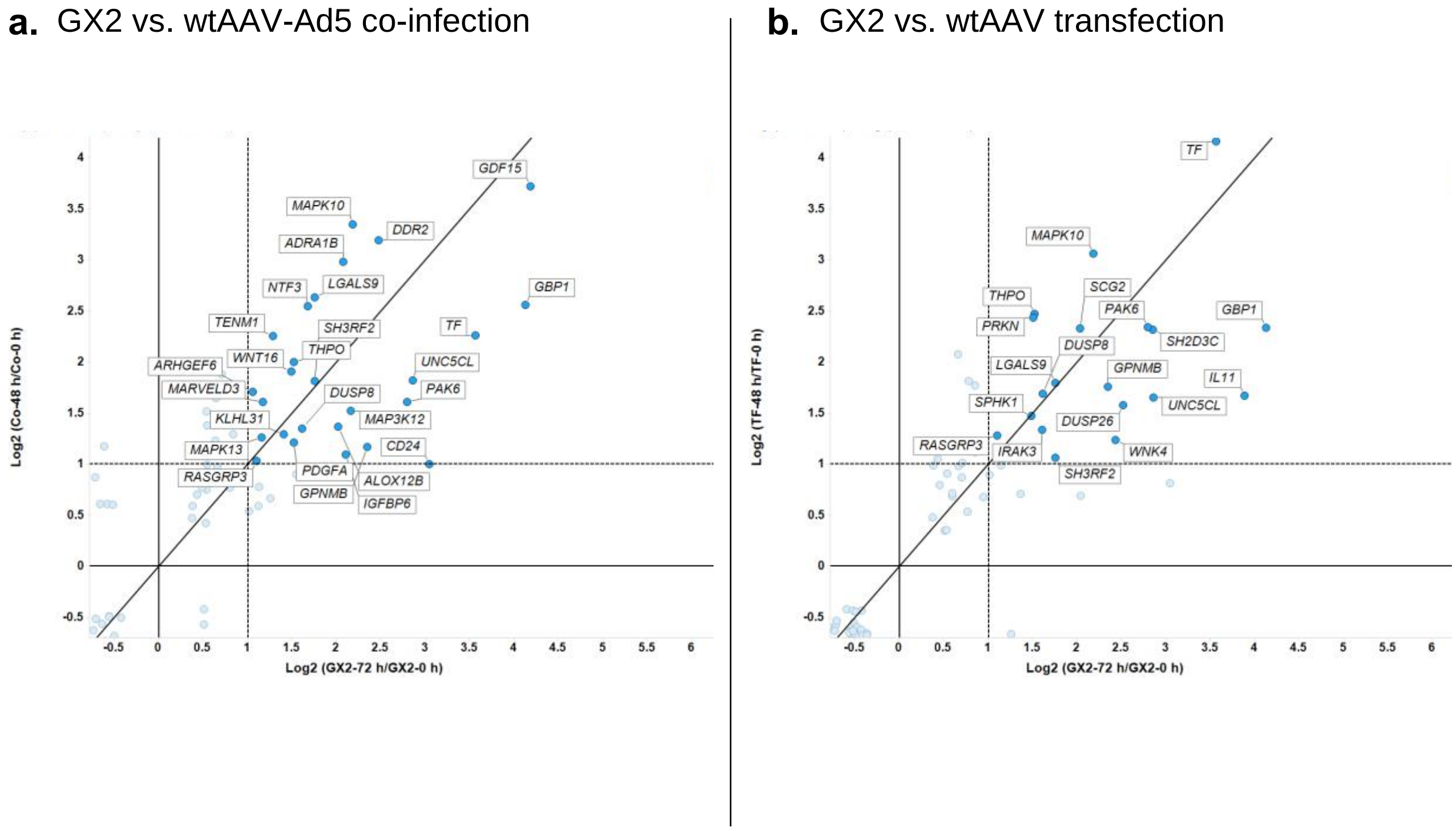

Supplemental Fig. S10. Transcript fold change of genes in the term MAPK signaling (GO:0000165) in three AAV systems. The fold change of all the MAPK signaling related genes were plotted and compared among three AAV cases. The fold change of antiviral genes upon production of wtAAV by co-infection ($\log_2$ (Co-48 hpi/Co-0 hpi)) (Case B) (a) and transfection ($\log_2$ (TF-48 hpi/TF-0 hpi) (Case C) (b) was compared to that by induction of GX2 ($\log_2$ (GX2-72 hpi/GX2-0 hpi) (Case A). The dash line in (a) and (b) mark a twofold change ($\log_2$ FC = 1). All labeled MAPK signaling-related genes had a FDR *p*-value below 0.05 in each comparison shown in the y-axis and x-axis titles of (a) and (b). *DUSP8* encodes dual specificity phosphatase 8, a key negative regulator of MAPK. Overexpression of *DUSP8* induces expression of the proapoptotic p53 upregulated modulator of apoptosis (PUMA), indicating the onset of programmed cell death (Lin et al. 2020). *GPNMB* encodes the transmembrane glycoprotein NMB, has been implicated in inflammation and neuroinflammation. Increased levels of *GPNMB* were commonly found in pro-inflammatory and pathological conditions (Saade et al. 2021). *SH3RF2* encodes a SH3-domain-containing RING finger protein, which is involved in promoting cell survival and apoptosis (Kim et al. 2014); *UNC5CL*, which encodes Unc-5 family C-terminal like protein, was identified as an inducer of a pro-inflammatory signaling cascade (Heinz et al. 2012). Across the transcript and protein levels, there were indications that functional classes related to inflammatory and immune responses were enriched (Cluster II in Fig. 4a and b). Together with the

upregulation of these MAPK signaling genes, the evidence for the occurrence of inflammation is stronger in all three cases.

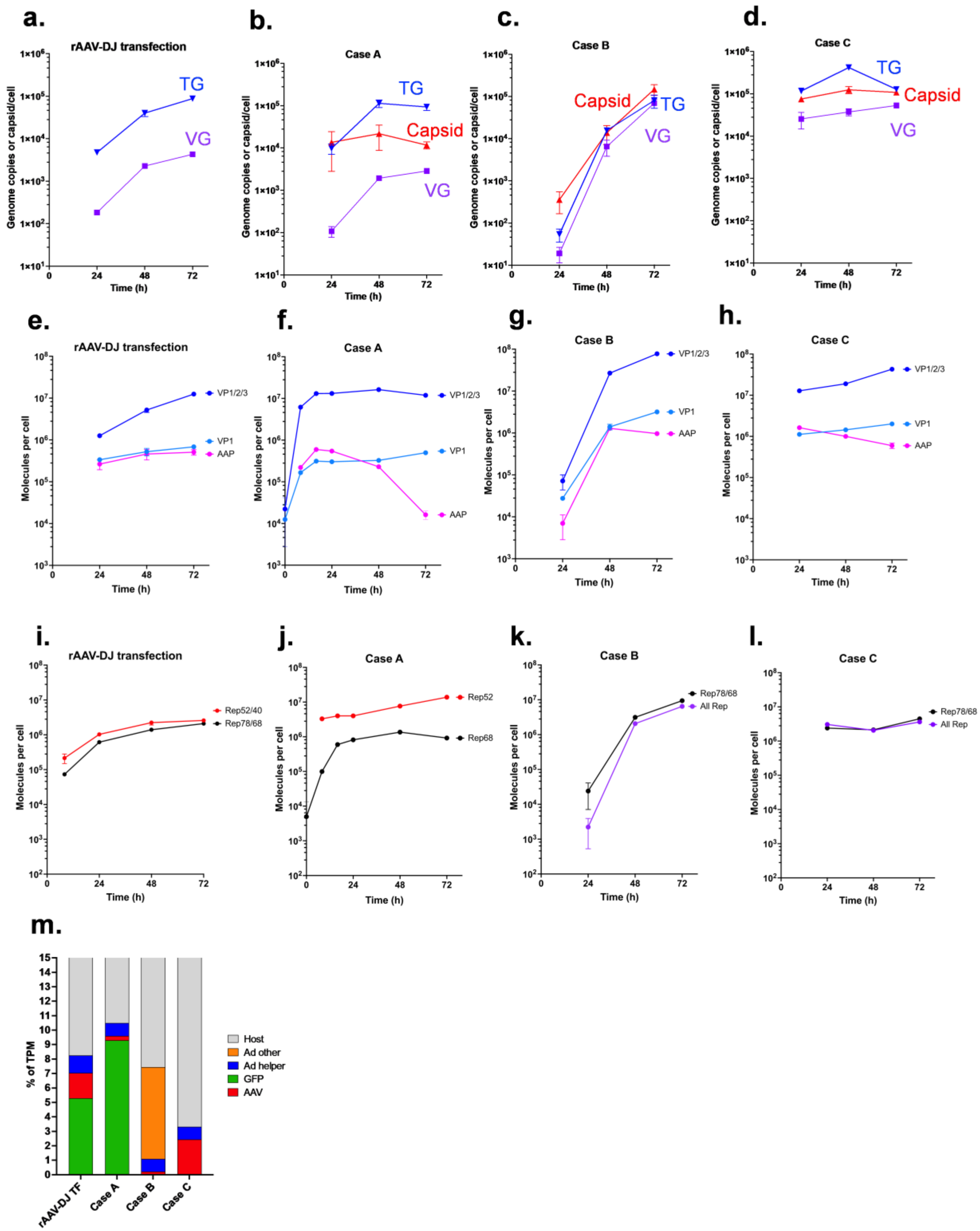

Supplemental Fig. S11. Time dynamics of viral components, transcripts and proteins in the production of AAV. (a, e, i): Recombinant AAV-DJ production by plasmid transfection of HEK293 cells (rAAV-DJ transfection),

(b, f, j) GX2 (Case A), (c, g, k): wtAAV-Ad5 co-infection (Case B), and (d, h, l): wtAAV transfection (Case C). VP123-Rep52 denotes the transcript encoding VP1/2/3 and Rep52 in GX2. VP23A denotes the truncated transcript encoding VP2, VP3 and AAP. (m) Percentage of transcripts of different categories of genes in the four AAV production systems at 48 h**.** The y-axis indicates the percentage (%) of total transcripts per million (TPM). "Ad other" represents the transcripts generated by all adenoviral genes except helper genes.

Supplemental Table S1. Primers used in qPCR assay.

Targeted Transcript	Sequence (5'-3')	References
GFP	F: TTCAAGGACGACGGCAACTAC<br>R: TCGATGCCCTTCAGCTCGAT	Lee et al. (2022)
AAV2 ITR	F: GGAACCCCTAGTGATGGAGTT<br>R: CGGCCTCAGTGAGCGA	Aurnhammer et al. (2012)
GPR15	F: GGTCCCTGGTGGCCTTAATT<br>R: TTGCTGGTAATGGGCACACA	Lu et al. (2024)

Supplemental Table S2. Sequences of heavy isotope-labeled peptides used in the targeted proteomics analysis (Lu et al. 2024).

Targeted Protein	Heavy isotope-labeled peptide sequence
EGFP	FSVSGEGEGDATYG[K_C13N15]
Rep78, Rep68	GIEPTLPNWFAVT[K_C13N15]
Rep78, Rep68, Rep52, Rep40	TAPDYLVGQQPVEDISSN[R_C13N15]
VP1, VP2, VP3	HPPPQILI[K_C13N15]
VP1	QLDSGDNPYL[K_C13N15]
AAP	APTEWVIP[R_C13N15]
DBP	NVSLPVAHSDA[R_C13N15]
Beta-actin (encoded by ACTB)	AGFAGDDAPR[R_C13N15]

Supplemental Table S3. Annotation of common mitochondrion-related DEGs in three AAV cases.

Gene Name	UniProt Accession (Protein)	Description	Average TPM					Log₂ (Relative protein abundance)		
			Host 293 (0 h)	Case A (Uni)	Case A (72 h)	Case B (48 h)	Case C (48 h)	Case A (72 h/Uni)	Case B (48 h/0 h)	Case C (48 h/0 h)
SREBF1		Sterol regulatory element binding transcription factor 1	17.0	34.9	18.0	37.4	7.2			
TIMM13	Q9Y5L4	Translocase of inner mitochondrial membrane 13	211.8	219.5	99.2	144.6	113.7	0.04	-0.35	-0.10
TSFM	P43897	Ts translation elongation factor, mitochondrial	65.0	70.2	32.8	51.4	49.9	-0.75*	0.02	-0.04
AARS2	Q5JTZ9	Alanyl-tRNA synthetase 2, mitochondrial	25.4	41.6	14.0	25.2	16.0	-0.84*	0.03	-0.07
MRPS26	Q9BYN8	Mitochondrial ribosomal protein S26	115.3	127.6	67.2	108.3	72.8	-0.38	0.11	-0.03
TIMM10B	Q9Y5J6	Translocase of inner mitochondrial membrane 10B	25.6	29.7	7.5	15.2	15.7	0.3	-0.03	0.02
PMPCA	Q10713	Peptidase, mitochondrial processing alpha subunit	60.1	116.4	54.1	57.0	37.3	-0.29	-0.06	-0.14
MRPL16		Mitochondrial ribosomal protein L16	64.2	77.2	33.8	58.6	44.3			
TRUB2		TruB pseudouridine synthase family member 2	23.6	32.3	11.5	15.1	15.3			
ACAD9	A0A7P0T871	Acyl-CoA dehydrogenase family member 9	20.9	37.8	14.3	27.2	12.2	-0.25	-0.05	-0.16
NDUFAF3	Q9BU61	NADH:ubiquinone oxidoreductase complex assembly factor 3	116.9	132.3	60.3	126.0	72.0	0.13	-0.08	-0.13
MRPL41	Q8IXM3	Mitochondrial ribosomal protein L41	104.3	111.4	63.1	48.4	56.9	-0.83*	-0.52*	-0.28
MT-ND6		Mitochondrially encoded NADH:ubiquinone oxidoreductase core subunit 6	547.3	299.8	80.3	257.7	149.4			
MT-ND2		Mitochondrially encoded NADH:ubiquinone oxidoreductase core subunit 2	5384.0	4992.4	1876.9	3947.4	2130.6			
MRPL28	Q13084	Mitochondrial ribosomal protein L28	139.4	171.3	72.1	81.7	116.7	-0.82*	-0.02	-0.13
NDUFB2		NADH:ubiquinone oxidoreductase subunit B2	709.6	516.7	162.8	248.0	644.1			
NDUFB7		NADH:ubiquinone oxidoreductase subunit B7	287.4	231.4	109.2	157.1	212.4			
TOMM22	Q9NS69	Translocase of outer mitochondrial membrane 22	158.3	197.9	84.0	93.2	142.4	0.49*	-0.16	-0.17
MRPL27	Q9P0M9	Mitochondrial ribosomal protein L27	212.7	120.2	52.7	76.5	181.1	-0.54*	-0.45*	-0.50*
MRPL3	E7ETU7	Mitochondrial ribosomal protein L3	185.9	160.0	51.2	96.8	198.0	-1.06*	-0.10	-0.03
MRPL15	Q9P015	Mitochondrial ribosomal protein L15	191.5	153.4	60.6	110.8	281.1	-0.65*	-0.01	0.01
NDUFC2	O95298	NADH:ubiquinone oxidoreductase subunit C2	840.9	647.7	133.8	400.6	735.4	-1.05*	-0.23	-0.38*

OMA1		OMA1 zinc metallopeptidase	20.3	20.4	4.0	16.3	20.4			
MRPL1	Q9BYD6	Mitochondrial ribosomal protein L1	44.4	26.2	7.1	28.9	105.1	-0.56*	-0.06	-0.08
GADD45GIP1	Q8TAE8	GADD45G interacting protein 1	89.4	62.1	26.3	39.4	114.4	-0.85*	0.06	0.09
MRPL14	Q6P1L8	Mitochondrial ribosomal protein L14	168.6	184.4	68.8	92.5	130.7	-1.02*	-0.16	-0.08
COA3	Q9Y2R0	Cytochrome c oxidase assembly factor 3	145.1	106.2	41.2	100.5	156.0	-0.86*	-0.41*	0.04
NDUFA12	Q9UI09	NADH:ubiquinone oxidoreductase subunit A12	273.9	237.6	112.1	180.7	338.1	-1.57*	-0.18	-0.28*
MRPL40	Q9NQ50	Mitochondrial ribosomal protein L40	108.1	80.9	27.5	56.1	139.6	-0.91*	-0.15	-0.21
NDUFAF8		NADH:ubiquinone oxidoreductase complex assembly factor 8	537.7	276.0	122.5	164.4	504.1			-0.10
MRPS17		Mitochondrial ribosomal protein S17	40.1	34.0	15.3	20.0	44.7	0.04*	-0.35	-0.04

"*" represents *p*-value below 0.05, and "Uni" indicates uninduced.

Supplemental Table S4. Annotation of common DEGs in GO: 0000165 (MAPK cascade) in the three AAV cases.

**Gene Name**	**Description**	**Average TPM**					
		Host 293 (0 h)	Case A (Uninduced)	Case A (48 h)	Case A (72 h)	Case B (48 h)	Case C (48 h)
THPO	Thrombopoietin	0.08	0.20	0.54	0.59	0.45	0.39
TF	Transferrin	0.05	0.26	1.66	1.71	0.26	1.00
MAPK10	Mitogen-activated protein kinase 10	0.16	0.87	1.56	2.39	2.18	1.23
GBP1	Guanylate binding protein 1	0.09	0.07	0.41	0.62	0.54	0.35
UNC5CL	Unc-5 family C-terminal like	0.48	0.59	1.93	4.31	1.60	1.08
GPNMB	Glycoprotein nmb	0.63	1.22	2.73	3.24	2.87	3.77
PAK6	p21 (RAC1) activated kinase 6	0.10	0.29	0.91	1.73	0.45	0.31
SH3RF2	SH3 domain containing ring finger 2	0.29	0.94	2.55	3.63	1.25	1.06
DUSP8	Dual specificity phosphatase 8	3.28	6.91	16.50	27.77	8.95	8.13

REFERENCES

Araujo FD, Stracker TH, Carson CT, Lee DV, Weitzman MD (2005) Adenovirus type 5 E4orf3 protein targets the Mre11 complex to cytoplasmic aggresomes. J Virol 79:11382-11391. doi: 10.1128/JVI.79.17.11382-11391.2005

Aurnhammer C, Haase M, Muether N, Hausl M, Rauschhuber C, Huber I, Nitschko H, Busch U, Sing A, Ehrhardt A, Baiker A (2012) Universal real-time PCR for the detection and quantification of adeno-associated virus serotype 2-derived inverted terminal repeat sequences. Hum Gene Ther Methods 23:18-28. doi: 10.1089/hgtb.2011.034

Debbas M, White E (1993) Wild-type p53 mediates apoptosis by E1A, which is inhibited by E1B. Genes Dev 7:546-554. doi: 10.1101/gad.7.4.546

Heinz LX, Rebsamen M, Rossi DC, Staehli F, Schroder K, Quadroni M, Gross O, Schneider P, Tschopp J (2012) The death domain-containing protein Unc5CL is a novel MyD88-independent activator of the pro-inflammatory IRAK signaling cascade. Cell Death Differ 19:722-731. doi: 10.1038/cdd.2011.147

Kim TW, Kang YK, Park ZY, Kim YH, Hong SW, Oh SJ, Sohn HA, Yang SJ, Jang YJ, Lee DC, Kim SY, Yoo HS, Kim E, Yeom YI, Park KC (2014) SH3RF2 functions as an oncogene by mediating PAK4 protein stability. Carcinogenesis 35:624-634. doi: 10.1093/carcin/bgt338

Lee Z, Lu M, Irfanullah E, Soukup M, Hu WS (2022) Construction of an rAAV producer cell line through synthetic biology. ACS Synth Biol 11:3285-3295. doi: 10.1021/acssynbio.2c00207

Lin KM, Lin SJ, Lin JH, Lin PY, Teng PL, Wu HE, Yeh TH, Wang YP, Chen MR, Tsai CH (2020) Dysregulation of dual-specificity phosphatases by epstein-barr virus LMP1 and its impact on lymphoblastoid cell line survival. Journal 94:e01837-19. doi: 10.1128/jvi.01837-19

Lu M, Lee Z, Lin YC, Irfanullah I, Cai W, Hu WS (2024) Enhancing the production of recombinant adeno-associated virus in synthetic cell lines through systematic characterization. Biotechnol Bioeng 121:341-354. doi: 10.1002/bit.28562

Saade M, de Souza GA, Scavone C, Kinoshita PF (2021) The role of GPNMB in inflammation. Front Immunol 12:674739. doi: 10.3389/fimmu.2021.674739

Schaley JE, Polonskaia M, Hearing P (2005) The adenovirus E4-6/7 protein directs nuclear localization of E2F-4 via an arginine-rich motif. J Virol 79:2301-2308. doi: 10.1128/JVI.79.4.2301-2308.2005

Shastri AA, Hegde V, Peddibhotla S, Feizy Z, Dhurandhar NV (2018) E4orf1: A protein for enhancing glucose uptake despite impaired proximal insulin signaling. PLoS One 13:e0208427. doi: 10.1371/journal.pone.0208427
